# Conservation and Diversity of Seed Associated Endophytes in *Zea* across Boundaries of Evolution, Ethnography and Ecology

**DOI:** 10.1371/journal.pone.0020396

**Published:** 2011-06-03

**Authors:** David Johnston-Monje, Manish N. Raizada

**Affiliations:** Department of Plant Agriculture, University of Guelph, Guelph, Ontario, Canada; Natural History Museum of Denmark, Denmark

## Abstract

Endophytes are non-pathogenic microbes living inside plants. We asked whether endophytic species were conserved in the agriculturally important plant genus *Zea* as it became domesticated from its wild ancestors (teosinte) to modern maize (corn) and moved from Mexico to Canada. Kernels from populations of four different teosintes and 10 different maize varieties were screened for endophytic bacteria by culturing, cloning and DNA fingerprinting using terminal restriction fragment length polymorphism (TRFLP) of 16S rDNA. Principle component analysis of TRFLP data showed that seed endophyte community composition varied in relation to plant host phylogeny. However, there was a core microbiota of endophytes that was conserved in *Zea* seeds across boundaries of evolution, ethnography and ecology. The majority of seed endophytes in the wild ancestor persist today in domesticated maize, though ancient selection against the hard fruitcase surrounding seeds may have altered the abundance of endophytes. Four TRFLP signals including two predicted to represent *Clostridium* and *Paenibacillus* species were conserved across all *Zea* genotypes, while culturing showed that *Enterobacter*, *Methylobacteria*, *Pantoea* and *Pseudomonas* species were widespread, with γ-proteobacteria being the prevalent class. Twenty-six different genera were cultured, and these were evaluated for their ability to stimulate plant growth, grow on nitrogen-free media, solubilize phosphate, sequester iron, secrete RNAse, antagonize pathogens, catabolize the precursor of ethylene, produce auxin and acetoin/butanediol. Of these traits, phosphate solubilization and production of acetoin/butanediol were the most commonly observed. An isolate from the giant Mexican landrace Mixteco, with 100% identity to *Burkholderia phytofirmans*, significantly promoted shoot potato biomass. GFP tagging and maize stem injection confirmed that several seed endophytes could spread systemically through the plant. One seed isolate, *Enterobacter asburiae*, was able to exit the root and colonize the rhizosphere. Conservation and diversity in *Zea*-microbe relationships are discussed in the context of ecology, crop domestication, selection and migration.

## Introduction

The first plants began colonizing land as early as 700 million years ago and like lichens, are hypothesized to have depended and co-evolved with microbes for stress tolerance and nutrient acquisition [1]. Endophytes are microbes that can be found living inside plant tissues, where they can live commensally or execute beneficial functions for the host [1]. It is likely that every plant species harbours endophytes, and indeed seeds of many plant species have been reported harbouring endophytes [2]–[4]. Plant seeds usually fall to the soil, a microbially rich habitat, and lie dormant waiting for environmental cues to germinate, possibly recruiting surface microbes to help protect against degradation or predation [5]. As seeds begin to germinate, seed endophytes may be important founders of the seedling microbial community as shown in rice [6], [7], eucalyptus [8] and maize [9]. Seeds are of particular interest as they may transmit endophytes vertically from generation to generation.

Seeds of the genus *Zea* have changed dramatically over time and this may have also dramatically altered associated microbial communities. Maize (*Zea mays* ssp. *mays*) is a giant grass that was domesticated from wild grasses (teosintes) about 9,000 years ago, in southwestern Mexico [10]. This process involved seed enlargement, elimination of the protective hard fruit case surrounding the seed, enhancement of husk leaves to protect an enlarged cob, development of non-shattering structures (rachids) to keep seed on the cob, the switching of seed placement on the plant and reduced shoot branching to permit greater nutrient allocation to seeds [11]. These changes so profoundly affected seed dispersal and germination that domesticated maize can no longer survive in the wild, intimately tying maize genetics to human selection and migration. While the wild teosinte relatives of maize are today mostly found in the mountains of Mexico and Central America, maize landraces were adapted by pre-Columbian peoples to diverse geographies including the temperate Gaspe Peninsula in Canada, the deserts of northern Mexico, the humid islands of the Caribbean, and the Andes mountains in South America [12], [13]. Numerous studies exist on the endophytes of maize [14]–[33] but these have not focused on seeds nor on the effects of human selection and migration on maize to new environments. Comprehensive studies in animal systems have shown that differences in mammal gut microbe populations correlate with host phylogeny rather than external animal environment [34], [35].

**Figure 1:**
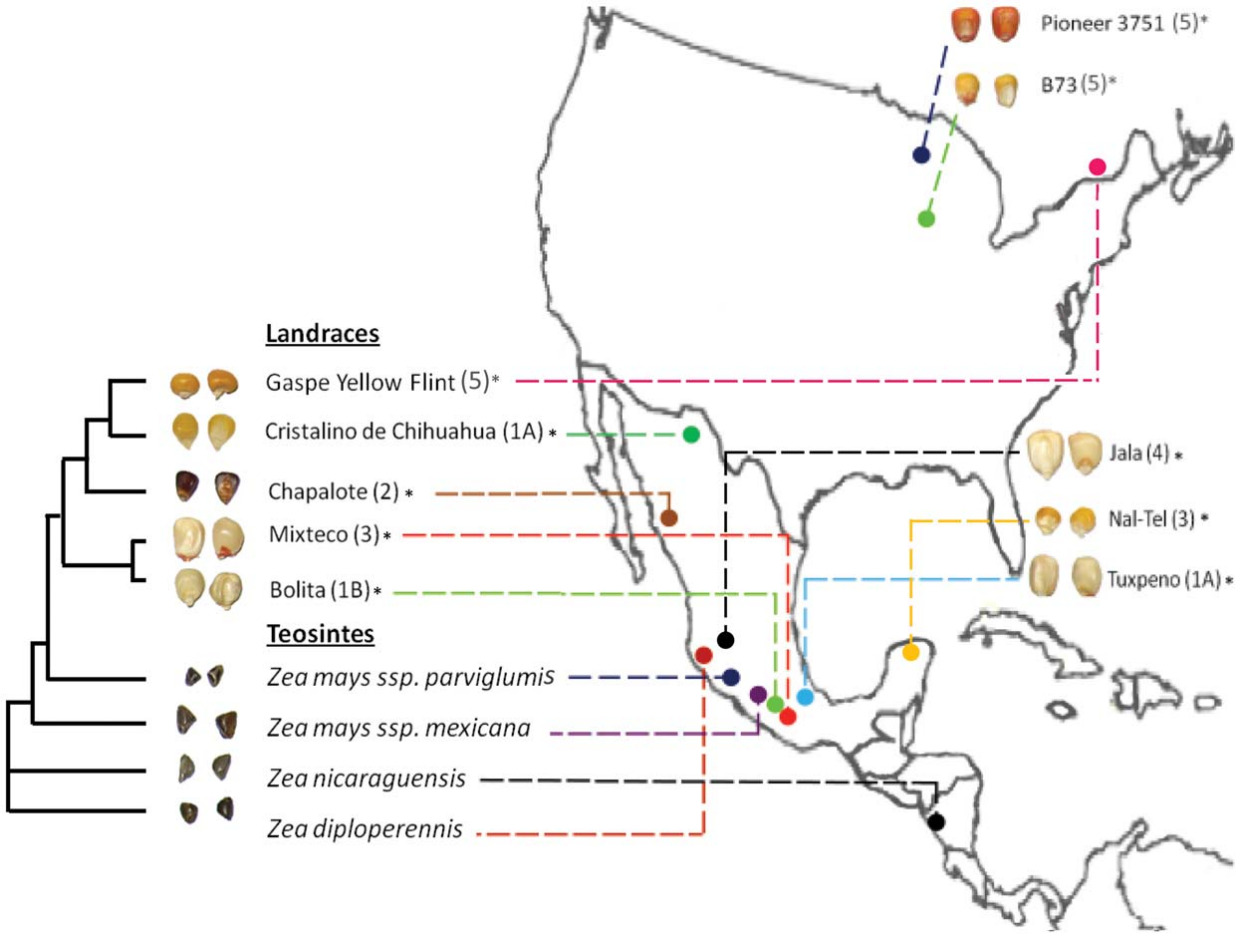
Genetic and geographic relationships of *Zea* genotypes used in this study. A microsatellite based dendogram shows the known genetic relationship between genotypes, (adapted from [13]), while dotted lines show where seed originate. Group 1 maize landraces grow in semi-hot temperatures of 14–21°C under either semi-dry conditions (540 to 640 mm)(1A) or semi-wet conditions (over 650 mm)(1B). Group 2 landraces grow in hot temperatures (20 to 27°C) and semi-wet growing seasons (500 to 870 mm of precipitation). Group 3 landraces grow in very hot (24.5–27.5°C) and wet (990–1360 mm) growing seasons. Group 4 plants are found mostly at mid elevations in Western Mexico (1200–1800 m) and form very large and numerous kernels. Group 5 plants are temperate landraces. The asterisk indicates seed used were not actually grown at this location; see Table 1 for details.

To study the effect of *Zea* evolution, genetic selection and migration on seed endophytes, a diversity panel of seeds were chosen based on their evolutionary relationships and adaptations to diverse environments (Figure 1 and Table 1). A *Zea* species microsatellite study [10] placed *Zea mays* ssp. *parviglumis* as the primary ancestor of domesticated corn, making Parviglumis of special evolutionary interest. Another teosinte from the mountains of Mexico, *Zea mays* ssp. *mexicana*, was included as it is thought to have contributed up to 12% of maize alleles [10]. Seeds of two other more divergent teosintes were included as outgroups, *Zea diploperennis*, a perennial relative of maize from the mountains of Jalisco, Mexico, and *Zea nicaraguensis* [36], an endangered, swamp inhabiting variety from Nicaragua. As Mexican maize landraces have been organized into four main ecological groupings [13], an effort was made to include one from each (Figure 1). Two maize landraces grown in the Mexican state of Oaxaca near the proposed area of corn domestication, Mixteco and Bolita, were included as examples of ancestral maize given their position at the base of the maize lineage [10]. Mixteco (similar to the more famous Oloton landrace) was of particular interest because of its giant stature under low nutrient conditions which has previously been speculated to be attributable to the activity of beneficial endophytes [37]. The large seeds of another giant maize plant, Jala, were included in the study as Jala is prized by local peoples in the Mexican state of Jalisco for having the largest cobs in the world (up to 36 cm long); the plants are grown on rich volcanic soils [38]. The maize landraces Chapalote (a northern Mexican popcorn) and Nal-Tel (a distinctive Yucatan variety found in ancient Mayan art) were included as they are considered to be “ancient indigenous varieties” [12] that may maintain ancestral microbial associations. As maize migrated northwards with humans, varieties were selected to adapt to new environments, resulting in new landraces such as the northern Mexican Cristalino de Chihuahua [10], and ultimately the Canadian landrace Gaspe Flint, a dwarf variety that flowers under the long day temperate conditions of its northern climate and matures early before the onset of frost. Two other temperate varieties were included which might show the effects of modern breeding on maize: Pioneer 3751 is an elite hybrid cultivar that was previously used for endophyte studies [14], while B73 is an inbred yellow dent variety from the North American corn belt that was recently used in the sequencing of the maize genome [39].

**Table 1:** Information about first generation seed origin, reason for selection and human use.

*Zea* Seed	Reason for Selection	Bank Accession Number	Site of Seed Collection (this study)	Seed Origin	Elevation (masl)	Latitude	Longtitude	Human Seed Use
*Zea mays* ssp. *parviglumis* (Balsas)	Direct ancestor of corn, microsatellite support	CIMMYT:11355	Km. 25 of Teleloapan-Arcelia Highway, Guerrero	Km. 25 of Teleloapan-Arcelia Highway, Guerrero	1800	18.24	−99.54	none
*Zea mays* ssp. *mexicana* (Chalco)	Contributed genetic material to maize, microsatellite support	CIMMYT:11386	San Antonio Tlatenco, Mexico, Mexico	San Antonio Tlatenco, Mexico, Mexico	2200	19.16	−98.55	none
*Zea nicaraguensis*	Swamp variety with flood tolerance mechanisms	CIMMYT: 11083 (Itlis 30919)	Chinandega, Nicaragua	Chinandega, Nicaragua	3	12.45	−87.05	none
*Zea diploperennis*	Corn relative with rhizomes and a distinct perenial lifestyle	CIMMYT:9476	Las Joyas, Cuatitlan, Jalisco	Las Joyas, Cuatitlan, Jalisco	1950	19.37	−104.12	none
Cristalino de Chihuahua	Short season maize, microsatellite support (Group 1A Environment)	CIMMYT:6862 (CHH 254)	CIMMYT, Mexico	La Junta, Chihuahua	1900	28.33	−107.28	*nixtamal* tortillas, atole (maize gruel)
Chapalote	Ancient variety with dark teosinte-like kernels, microsatellite support (Group 2 Environment)	CIMMYT:861 (SINA 2)	CIMMYT, Mexico	Culiacán, Sinaloa	61	24.86	−107.42	popcorn and pinole (course, spiced flour)
Nal-Tel	Ancient landrace from Oaxaca and Yucatan (Group 3 Environment)	CIMMYT:815 (YUCA 7)	CIMMYT, Mexico	Dzitás, Yucatán	30	20.51	−88.31	popcorn, botanas (snacks)
Mixteco (Oloton)	Giant pre-Colombian exotic grown in Oaxaca on acidic, nutrient poor soil, microsatellite support (Group 3 Environment)	CIMMYT:24143 (OAX 569)	CIMMYT, Mexico	Magdalena Yodocono de Porfirio Díaz, Oaxaca	2500	17.38	−97.33	tortillas
Bolita	Modern incipient landrace, microsatellite support (Group 1B Environment)	CIMMYT:10503 (OAX 68)	CIMMYT, Mexico	Nochixtlan, Oaxaca	1645	17.29	−97.14	large, thin tortillas called *tlayuda*
Jala	Giant Species with giant kobs (Group 4 Environment)	CIMMYT:2215 (JALI 69)	CIMMYT, Mexico	Tlaqupaque, Jalisco	1616	20.40	−103.12	tortillas, botanas (snacks)
Tuxpeno	Landrace very important in CIMMYT breeding programs (Group 1A Environment)	CIMMYT:2536 (OAXA 9)	CIMMYT, Mexico	Tuxtepec, Oaxaca	91	18.07	−96.06	tortillas, botanas (snacks)
Gaspe Yellow Flint	Fast growing, dwarf, Canadian landrace, microsatellite support (Temperate Environment)	NCPRIS: Pi 214279	Castana, Iowa, USA	Montreal, Quebec, Canada	15	45.41	−73.94	cornmeal, corncake, soups
B73	American dent inbred, subject of corn genome sequencing efforts (Temperate Environment)	CIMMYT: 23811 (Pi 550473)	CIMMYT, Mexico	Castana, Iowa, USA	350	42.15	−95.84	livestock feed, chemical production
Pioneer 3751 (130 MaximXL, ApronXL)	Commercial hybrid used in corn root endophyte study (Temperate Environment)	14498792 (T3SZZA11015.00)	Szarvas, Hungary	Minnesota, USA	unknown	unknown	unknown	livestock feed, chemical production

In this study we investigated interactions between *Zea* genotype, seed source, and endophytic microbiota using culturing and culture independant methods to evaluate both microbial diversity and activity. We found that seed bacterial communities varied with *Zea* phylogeny, but there was also a core microbiota that was conserved across *Zea* genotypes.

## Results

### Seed endophytes reflect *Zea* phylogenetic relationships

**Figure 2:**
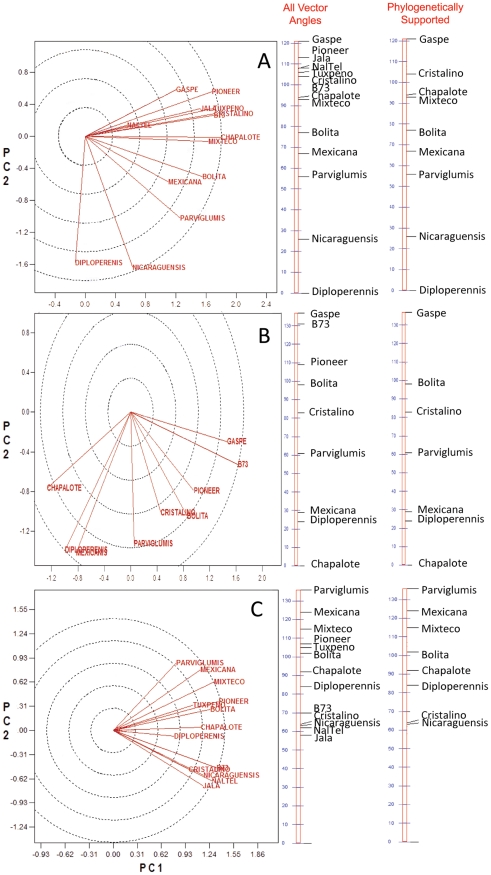
Biplot diagrams to show the relatedness of endophytic microbial communities between *Zea* genotypes. The analysis is based on principle component analysis (PCA) of bacterial 16S rDNA terminal fragment length polymorphism (TRFLP) fingerprints. Shown are PCA analysis for (A) first generation seeds, (B) second generation seeds, and (C) stems. Vectors are drawn in red and represent the different *Zea* genotype samples. Diagrams are biplots of the first and second principle components, and are based on covariance between samples for differently sized forward and reverse terminal fragments (not shown). Angles between vectors represent the degree of covariance between samples, and are summarized on the vertical bars next to each biplot. The genotypes Nal-Tel, Tuxpeno, Jala, Pioneer 3751, and B73, lack phylogenetic support in this study, so they are omitted from vector angle bars on the right.

PCA analysis of covariance was performed on seed and stem 16S rDNA TRFLP results (Table S1) to statistically evaluate if endophyte community composition reflects evolutionary and/or ecological relationships of their host plants (Figure 2). Endophytes from first generation seed obtained from plants growing in different geographic environments show a striking pattern of covariation that recapitulates the phylogenetic pattern of their *Zea* hosts (Figure 1, 2A). Endophytes from distinct *Zea* species (Diploperennis and Nicaraguensis) were placed at one end of the spectrum, followed by the direct ancestors of modern maize (Parviglumis and Mexicana), then ancestral subspecies (Bolita and Mixteco), terminating in more derived (Chapalote and Cristalino) and temperate landraces (Gaspe). Mature second generation seed obtained from plants growing in the same temperate field were obtained from only a subset of genotypes, and their endophytes showed a similar pattern of covariation to first generation seed except that Chapalote appeared as an outlier (Figure 2B). Unlike seeds, endophytes in second generation stems appeared random with respect to *Zea* evolution (Figure 2C).

With respect to the ecology of *Zea mays* hosts, temperate seeds (Gaspe, Pioneer, B73) but not stems tended to harbour distinct endophytic communities compared to tropical plant genotypes (all others)(Figure 2A, B). Generation 2 seed was of special interest as it maintained the temperate and tropical groupings in spite of the parent plants being grown in the same temperate environment (Figure 2B). Within the tropical *Zea mays* varieties, no patterns based on the ecological groupings of the host plants was observed. For example, in both Generation 1 seed and Generation 2 seeds, endophytes from the two extreme ecological zones (Zone 1A, Tuxpeno, Cristalino; Zone 3, Nal Tel, Mixteco) did not form distinct groups (Figure 2A, B).

### There is conservation of seed endophytes in *Zea* across boundaries of evolution, ethnography and ecology

**Figure 3:**
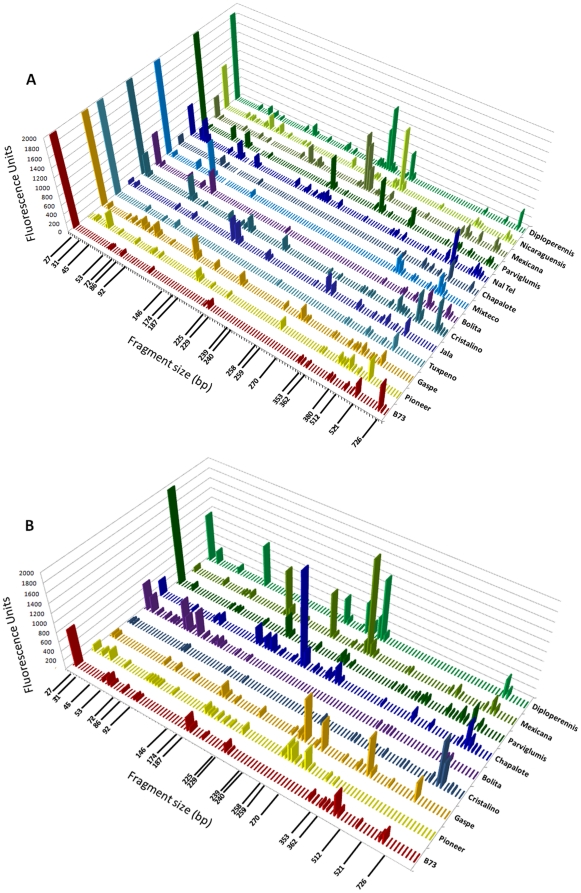
Profiles of endophytic microbial communities present in seeds of diverse *Zea* genotypes. Shown are profiles of (A) first generation seeds and (B) second generation seeds using bacterial DNA fingerprinting (16S rDNA TRFLP). Each peak is the fluorescence intensity average of 3 TRFLP amplifications from 15 pooled seed, a semi-quantitative indicator of microbial titre. The immediate parents of Generation 1 seeds were grown in diverse geographic locations as indicated in Figure 1 and Table 1, while all Generation 2 seeds came from Generation 1 seeds planted in a common field in Guelph, Canada. 16S rDNA amplicons were first generated using forward primers 799f/1492rh and then were restricted using *Dde*I. Small fragments and those corresponding to 16S chloroplast rDNA or 18S rDNA were removed. In Generation 2, a few genotypes did not produce mature seed and were not included.

**Figure 4:**
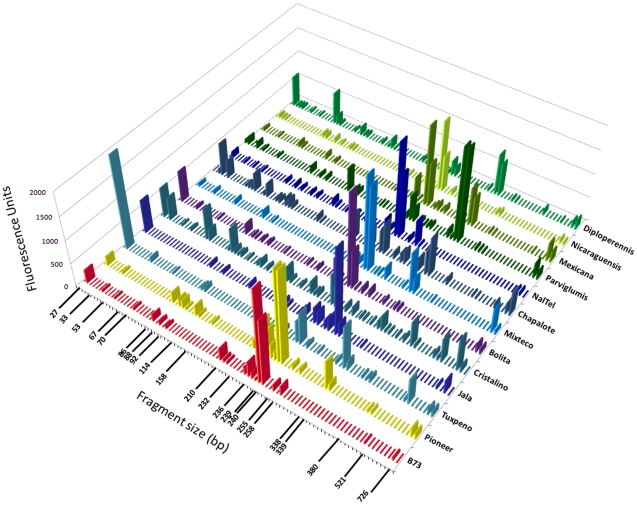
Profiles of endophytic microbial communities present in stems of diverse *Zea* genotypes using bacterial DNA fingerprinting (16S rDNA TRFLP). Each peak is the fluorescence intensity average of 3 TRFLP amplifications from 10 pooled samples, a semi-quantitative indicator of microbial titre. All samples were taken from basal stem region of plants of Generation 1 seeds grown in a common field in Guelph, Canada. 16S rDNA amplicons were generated using forward primers 799f/1492rh followed by restriction using *Dde*I. Small fragments and those corresponding to 16S chloroplast rDNA or 18S rDNA were removed. Landrace Gaspe Flint stems died before harvest and were not included.

**Figure 5:**
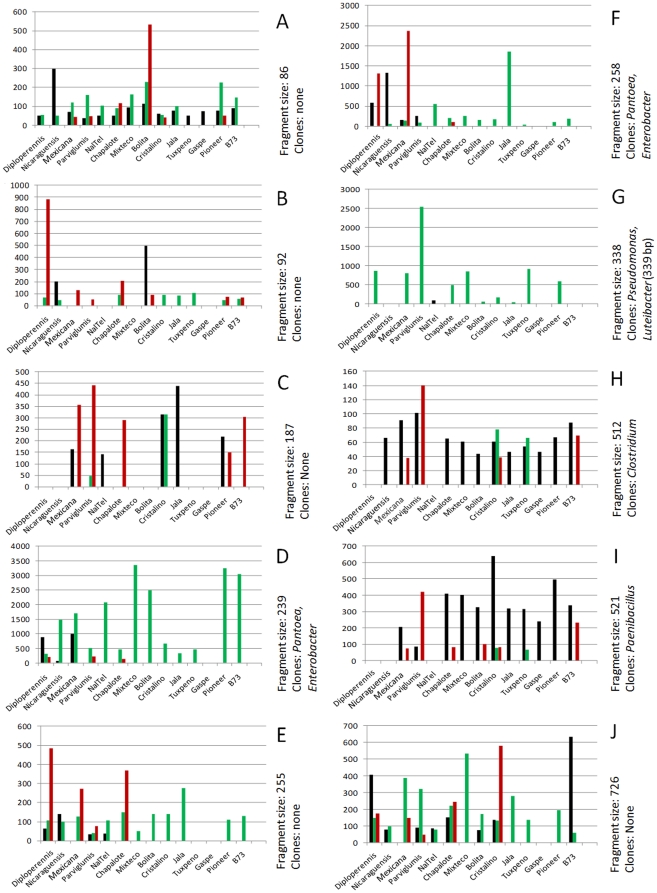
Select *Zea* seed endophyte TRFLP profiles demonstrating the range of inheritance patterns observed across samples. Each panel shows the TRFLP fluorescence intensities for a given bacterial 16S rDNA forward size fragment in pooled Generation 1 seeds (black), their pooled stems (green) and subsequent pooled Generation 2 self/sib pollinated seeds (red). Corresponding 16S rDNA clones were sequenced and the predicted taxonomic identifications are indicated. Shown are transmission patterns for the following TRFLP forward size fragments: (A) 86 bp, (B) 92 bp, (C) 187 bp, (D) 239 bp, (E) 255 bp, (F) 258 bp, (G) 338 bp, (H) 512 bp, (I) 521 bp, (J) 726 bp.

**Figure 6:**
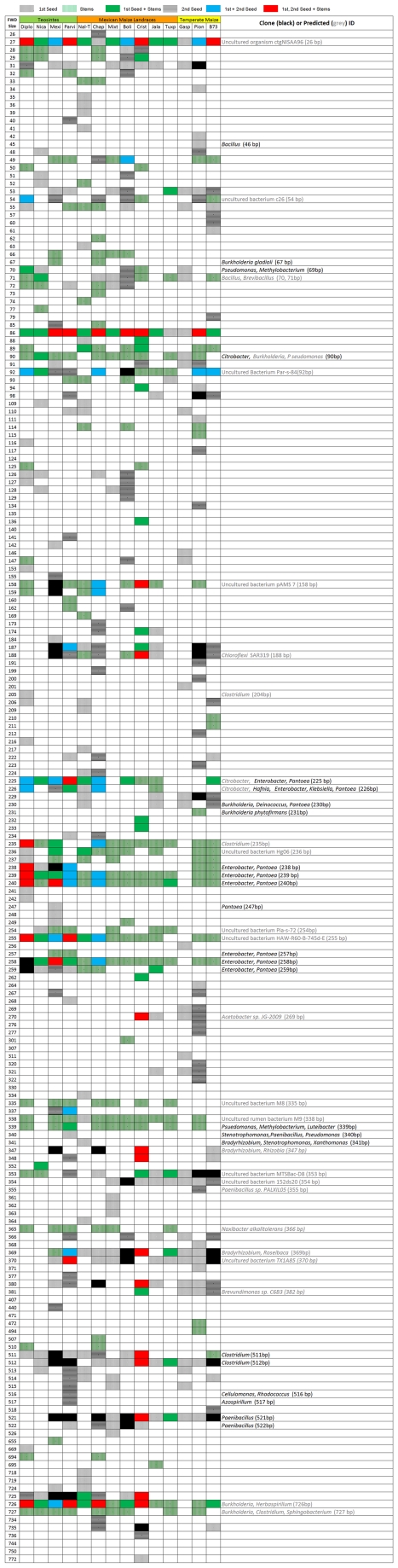
Summary of forward labelled 16S rDNA TRFLP fragments in seeds and stems displayed as presence or absence data. Fragment sizes are listed on the left side in base pairs, and fragments are noted as being present if amplified in at least 1 of the 3 PCR trials but not the water control. Potential fragment identities were determined by sequencing of isolates or clones, or by submitting raw TRFLP data to APLAUS+. Microbial presence is indicated by coloured shading depending on which plant samples it was observed in, with grey being Generation 1 seed, horizontal black bars being Generation 2 seed, vertical green bars being stem tissue, black being Generation 1 and 2 seed, green being Generation 1 seed and stems, blue being Generation 2 seed and stems, and red being Generation 1 and 2 seed plus stems. Fragments smaller than 25 bp and those representing mitochondrial 18S (536–538 bp) were excluded from the display.

**Figure 7:**
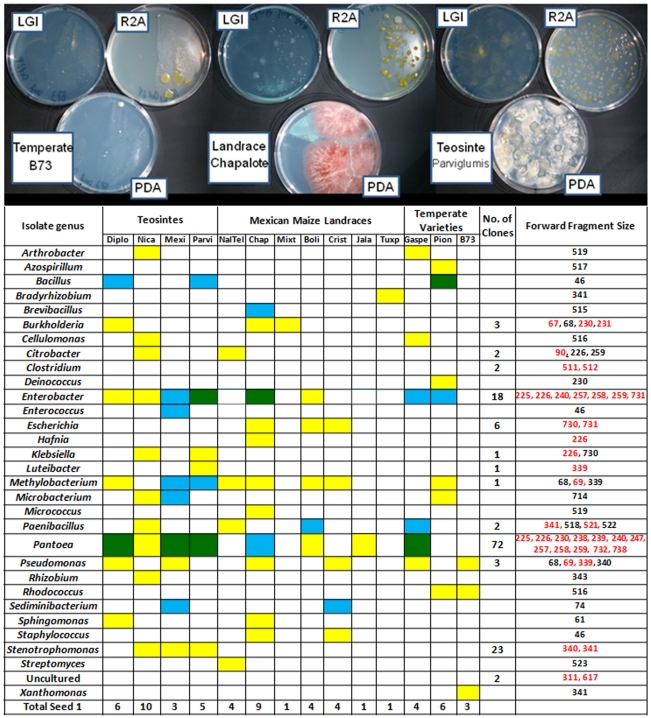
Examples of microbes cultured on diverse media (LGI, R2A, and PDA) from *Zea* seed pools followed by genus level taxonomic identification of all unique colonies. For each genus (row), a yellow box indicates successful culturing of that genus from Generation 1 seed, blue indicates culturing from Generation 2 seed, and green indicates culturing from both generations. Taxonomic identification was based on sequencing of 16S rDNA. Predicted 16S rDNA *Dde*I forward cleavage product fragment sizes are indicated for each genus from both cultures (black text) and from PCR clone libraries (red text).

The Generation 1 seed came from a diversity of *Zea* species and subspecies from parents grown in different geographic locations across North America and Europe (Table 1). In Generation 2, these seed all came from parents growing in a new field in Canada. In spite of these differences, TRFLP analysis showed that there were four 16S peaks conserved across *Zea* groups in both Generation 1 and Generation 2 seeds (Figure 3A, 3B). Two of these peaks with 512 and 521 bp sizes were not often observed in stem tissue (Figure 4, 5). Cultured isolates and 16S PCR clones from seed DNA were sequenced and used to predict taxonomic identities which were then matched *in silico* to TRFLP fragment sizes (Figure 6, 7). Sequences were submitted to Genbank and received accession numbers JF753273–JF753552. When neither clone nor culture 16S information was available, forward and reverse TRFLP fragments (Table S1) were submitted to the APLAUS+ bacterial TRFLP prediction program to predict microbial identity [40]. The peak sizes of the conserved set and their predicted taxonomic identities were: 27 bp (unidentified), 86 bp (unidentified), 511/512 bp (99% to *Clostridium beijerinckii*) and 521 bp (100% to *Paenibacillus* sp. IHB B 2257). Their frequencies amongst *Zea* genotypes were as follows: 27 bp (Seed 1, 11/14 *Zea* genotypes; Seed 2, 7/9 *Zea* genotypes), 86 bp (Seed 1, 14/14; Seed 2, 6/9)(Figure 5A), 511/512 bp (Seed 1, 14/14; Seed 2, 5/9)(Figure 5H) and 521 bp (Seed 1, 11/14; Seed 2, 6/9)(Figure 5I). TRFLP peak 726 bp was also conserved across *Zea* subgroups in Generation 1 seed (8/14) and stems (13/13) but less so in Generation 2 seed (5/13)(Figure 5J). Peak 726 bp is predicted to represent *Burkholderia* or *Herbaspirillum* spp. based on APLAUS+. Culturing also showed that *Methylobacteria*, *Pantoea* and *Pseudomonas* were conserved across all *Zea* groups in Generation 1 seed, while only *Enterobacter* species were isolated from all groups of *Zea* seed in Generation 2 (Figure 7); there was also a predicted *Methylobacteria/Psuedomonas* TRFLP peak (338/339 bp) which was conserved in stems (but not seeds) across *Zea* genotypes (Figure 4, 5G). We conclude based on TRFLP evidence that there is a heritable seed core microbiota in *Zea* that is conserved across boundaries of evolution, human selection and ecology.

### Seed migration may affect seed endophyte communities

The Generation 1 to Generation 2 migration experiment mimicked modern breeding in which crop seeds are routinely moved around the world. In domesticated maize (n = 6), the percentage of TRFLP peaks observed in Generation 1 seed that persisted in Generation 2 seed following migration was 13–22% with the exception of Cristalino which was 44% (Figure 3A, 3B, 6). There were also differences in the culturable bacteria between generation 1 and 2 seed (Figure 7), with only 9 genera observed in generation 2 seed as opposed to 26 in generation 1 seed. Although PCA analysis suggested that the effect of external environment was less important than plant genotype, these observations suggest that only a fraction of seed endophytes persist during migration associated with modern breeding.

### There is a highly conserved stem microbiota across *Zea*

Stems base samples had TRFLP peaks that were shared across *Zea* subgroups including 27 bp (present in 12/13 *Zea* genotypes), 86 bp (12/13), 89/90 bp (12/13), 92/93 bp (11/13), 158/159 bp (8/13), 225/226 bp (10/13), 235/236 bp (12/13), 239/240 bp (13/13), 254/255 bp (13/13), 258/259 bp (12/13), 338/339 bp (10/13), 726 bp (13/13)(Figure 4, 6). The predicted taxonomic identities were as follows: 27 bp (unidentified), 86 bp (unidentified), 89/90 bp (97% to *Citrobacter freundii*), 92/93 bp (uncultured bacterium), 158/159 bp (uncultured bacterium), 225/226 bp (*Hafnia, Enterobacter, Klebsiella, or Pantoea* species), 235/236 bp (predicted to be an uncultured *Clostridium* sp.), 239/240 bp (*Enterobacter* and *Pantoea* species), 254/255 bp (uncultured bacterium), 258/259 bp (*Enterobacter* and *Pantoea* species), 338/339 bp (*Psuedomonas*, *Methylobacterium* and *Luteibacter* species), 726 bp (predicted to be *Burkholderia/Herbaspirillum* species)(Figures 4, 5; Tables S2, S3). This high conservation might explain the lack of groupings based on *Zea* phylogeny in the PCA analysis (Figure 2C).

### Endophyte communities in different plant tissues are distinct

We asked whether there were distinct microbiotas in seeds compared to stem tissues. There was no significant difference in the number of TRFLP peaks observed in seeds versus stems integrating all genotypes (p>0.05, Mann-Whitney). However, of TRFLP peaks observed in Generation 1 and/or 2 seeds in more than one genotype, 47% (26/55) were not found in stem tissue (Figure 3, 4, 6). Of TRFLP peaks observed in stems of more than one genotype, 29% (11/38) were not observed in seeds. Included in the unique seed microbiota were a 31 bp peak, a 229/230 bp peak (predicted to be *Burkholderia phytofirmans* or *Pantoea*) and peaks 513–515 bp. The unique stem community included a 335 bp peak, a 365 bp peak and a 727 bp peak (genus *Burkholderia*, *Clostridium* or *Sphingobacterium*, based on APLAUS+); no bacteria having predicted TRFLP peaks of this size were cultured from seeds (Figure 7). This data suggests that different plant tissues can have distinctive microbiota.

### There was no significant difference in the amount of endophytic diversity observed in wild versus domesticated *Zea* species

We asked whether there was a greater diversity of endophytes in wild teosinte plants than their domesticated counterparts. There was no significant difference in the number of TRFLP peaks in either Generation 1 seeds or stems comparing teosinte (n = 4) to domesticated maize (n = 10)(p = 0.41, t-test). There was also no significant difference in the number genera of microbes cultured in Generation 1 seeds between these groups (P = 0.16, t-test).

### There are *Zea* species-specific endophytes

A host speciation barrier might prevent exchange of seed endophytes and allow for selection of unique microbiotas. All plant genotypes used in this study belonged to the species *Z. mays* except for *Z. diploperennis* and *Z. nicaraguensis*. Three TRFLP peaks were found at a significantly higher frequency in the *Z.mays* subgroup (n = 20) than the non-*Z. mays* group (n = 3)(P<0.05, Fisher's Exact Test): 187/188 bp (*Chloroflexi* based on APLAUS+ prediction)(Figure 4C), 369/370 bp (*Bradyrhizobium* or uncultured bacterium TX1A85 based on APLAUS+) and 521 bp (99% to *Paenibacillus caespitis*)(Figure 7; Tables S2, S3). These peaks were not found in any *Z. diploperennis* or *Z. nicaraguensis* generation 1 or 2 seeds or stems, were not culturable, and were not found in clone sequence libraries (Figure 3, 4, 6; Tables S2, S3). In contrast, their frequencies of occurrence in Generation 1 and 2 seeds belonging to *Z. mays* genotypes were high: 187/188 bp (13/20 PCR attempts), 369/370 bp (15/20) and 521 bp (17/20)(Figure 5I). Reciprocally, seven TRFLP seed peaks were unique to *Z. diploperennis* and one to *Z. nicaraguensis* (Figure 3, 6).

### Endophytes in the wild ancestor persist in domesticated maize

The closest wild relatives of domesticated maize are *Z. mays* ssp. *parviglumis* and ssp. *mexicana*. We asked what percentage of endophytes found in these genotypes persist in any of the domesticated progeny (*Z. mays* ssp. *mays*) included in this study. Interestingly, 76% of TRFLP peaks observed in Parviglumis seeds, and 79% in stems were observed in at least one domesticated maize genotype. For Mexicana, 78% and 92% of seed and stem endophytes, respectively, were retained in at least one domesticated maize genotype. In terms of culturing, 4/7 and 6/7 genera of seed endophytes in Parviglumis and Mexicana, were also found in at least one domesticated maize (Figure 7). This data cannot distinguish ancestral endophytes that persisted during domestication versus endophytes that were coincidentally gained by Parviglumis/Mexica and/or modern maize after domestication. In either scenario however there is no evidence for a major selection sweep for endophytes caused by maize domestication, for example no evidence that host specificity was drastically altered genetically. In spite of this apparent lack of a selective sweep, neither *Klebsiella* nor *Stenotrophomonas*, genera found in both Parviglumis and Mexicana, were ever cultured in any of the 10 domesticated genotypes (Figure 7).

### Loss of the fruitcase during crop domestication may have altered the abundance of specific seed endophytes

During maize domestication, a protective, outer fruitcase and leafy glume were selected against by ancient peoples to permit processing of the seed (Figure 1) [41]. We asked if there was any difference in the microbiotas of seeds with fruitcases (Diploperennis, Nicaraguensis, Mexicana, Parviglumis) or without fruitcases (all others). In terms of frequency, three TRFLP fragment groups were significantly over-represented (P<0.005, Fisher's Exact Test) in *Zea* species with fruitcases (n = 7) than those without (n = 16)(Figure 3A, 3B, 6). These were peaks 238/239/240 bp and 258/259 bp (both predicted to be *Enterobacter* and/or *Pantoea* based on 16S rDNA sequencing) and peak 255 bp (predicted by APLAUS+ to be uncultured *Methanogen* HAW-R60-B-745d-E) [42]. The corresponding TRFLP peaks in teosinte were some of the largest observed in any seeds (Figure 3A, 3B). Domestication may have caused reductions in the titre of these bacteria in seeds, but not their elimination, as they were culturable from domesticated maize seeds (Figure 7) and were detectable in stems by TRFLP profiling (Figure 4).

### Most *Zea* genotypes possess endophytes that solubilize phosphate, secrete acetoin and may fix nitrogen

**Figure 8:**
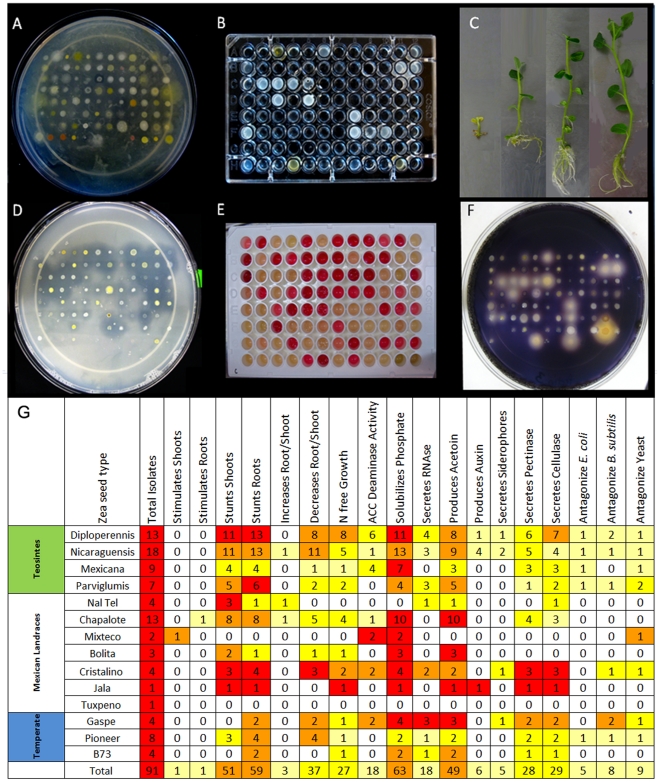
Analysis of functional traits of endophytes cultured from *Zea* seed. Shown are (A–F) select examples of trait assays and (G) the complete summary grouped by *Zea* genotype. Shown are assays for (A) antagonism to *E. coli*; (B) growth in nitrogren free LGI media with only ACC as a nitrogen source; (C) growth promotion of tissue cultured potato one month after inoculation with (from L–R) *Enterobacter cloacae*, *Cellulomonas denverensis*, sterile buffer, or *Burkholderia phytofirmans*; (D) ability to solubilise tricalcium phosphate; (E) acetoin and butanediol production; and (F) extracellular digestion of cellulose. For panel (G), light yellow shading indicates that <25% of isolates from the *Zea* genotype indicated exhibited the trait, deep yellow indicates 25–50%, orange indicates 50–75%, and red indicates 75–100%.

The above endophytic traits were ranked based on how widespread they were amongst *Zea* genotypes. The most common endophytic traits were phosphate solubilization (found in 12/14 *Zea* genotypes), followed by acetoin production (12/14), cellulase and/or pectinase secretion (12/14) and growth on nitrogen-free media (11/14)(Figure 8G; Table S4). Moderately conserved endophytic traits found across *Zea* subgroups were ACC deaminase activity (8/14), antibiosis against bacteria (8/14) or yeast (8/14) and RNase secretion (8/14). Rare endophytic traits were auxin production (3/14) and siderophore secretion (3/14).

**Figure 9:**
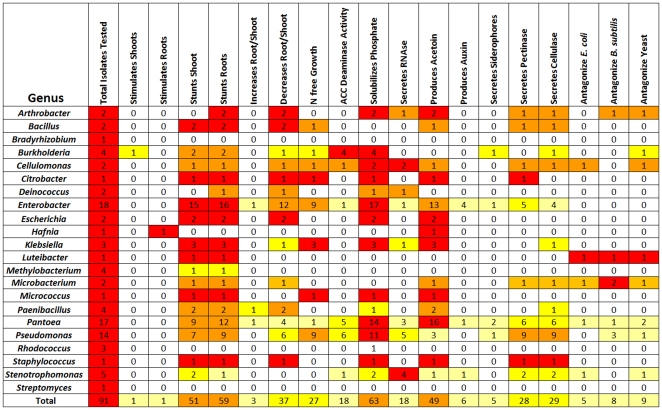
Summary of functional traits exhibited by cultured seed endophytes grouped by bacterial genus. Light yellow shading indicates that <25% of isolates from the *Zea* genotype indicated exhibited the trait, deep yellow indicates 25–50%, orange indicates 50–75%, and red indicates 75–100%. A more detailed listing by isolate is included in Table S4.

Interestingly three genera of microbes which were widely culturable across *Zea* (Figure 7), *Enterobacter*, *Pantoea* and *Pseudomonas*, were found to significantly contribute to the most conserved endophytic traits (Figure 9). Of 63 isolates that solubilised phosphate, 51 belonged to these genera. Similarly, 28/49 acetoin producers belonged to *Enterobacter* and *Pantoea*. Finally, 18/27 bacteria that could grow on nitrogen-free media belonged to *Enterobacter* and *Pseudomonas*.

### *Zea* endophytes can cause organ growth promotion or increase the root∶shoot biomass allocation in potato

A previously reported plant bioassay was used to test the ability of the endophytes to stunt or promote plant growth using inoculated tissue cultures of potato (*Solanum tuberosum* cv. Kennebec) [43]. Endophytes from most *Zea* genotypes stunted growth of potato shoots (51/91) or roots (59/91), but two microbial isolates significantly stimulated potato biomass accumulation of shoots or roots (Figure 8C, 8G). The shoot growth promoting microbe was cultured from surface sterilized seed of the maize landrace Mixteco and was identified as *Burkholderia phytofirmans* PSjN (100%); it increased shoot biomass significantly (Mann-Whitney-Wilcoxon, p = 0.009) by ∼70% compared to the buffer control (Figure 8C, 8G; Table S4). Mixteco is of interest because it has a giant shoot [37] raising the possibility that *B. phytofirmans* contributes to this trait. The other giant maize genotype in this study was Jala [38]. The sole cultivatable seed endophyte from Jala was *Pantoea ananatis* (99%) which was the only *Pantoea* able to grow on low nitrogen LGI media (Figure 9) suggestive of N-fixation activity. *P. ananatis* was also the only endophyte in any *Z. mays* genotype to produce auxin (Figure 9), a trait associated with root growth [21]. *P. ananatis* did not however cause potato growth promotion (Figure 9). The potato root growth promoting strain was *Hafnia alvei* (100%) from Chapalote seeds; it increased root biomass significantly (Mann-Whitney-Wilcoxon, p = 0.036) by ∼2-fold compared to the buffer control (Figure 9; Table S4).

Plants can adapt to low nutrient stress by decreasing their biomass allocation to shoots, while maintaining root biomass, resulting in a higher root∶shoot biomass ratio [44]. This strategy maintains nutrient scavenging by roots, but with less nutrient requirements for shoot growth. We found that a single microbe, *Paenibacillus caespitis*, from Nal-tel seeds, significantly reduced potato shoot biomass (Mann-Whitney-Wilcoxon, p = 0.036) without significantly reducing the root biomass (Mann-Whitney-Wilcoxon, p>0.999), nearly doubling the root∶shoot ratio (0.41) compared to the buffer control (0.23) (Figure 9; Table S4).

### GFP tagged microbes can be observed in vascular tissue and rhizosphere

**Figure 10:**
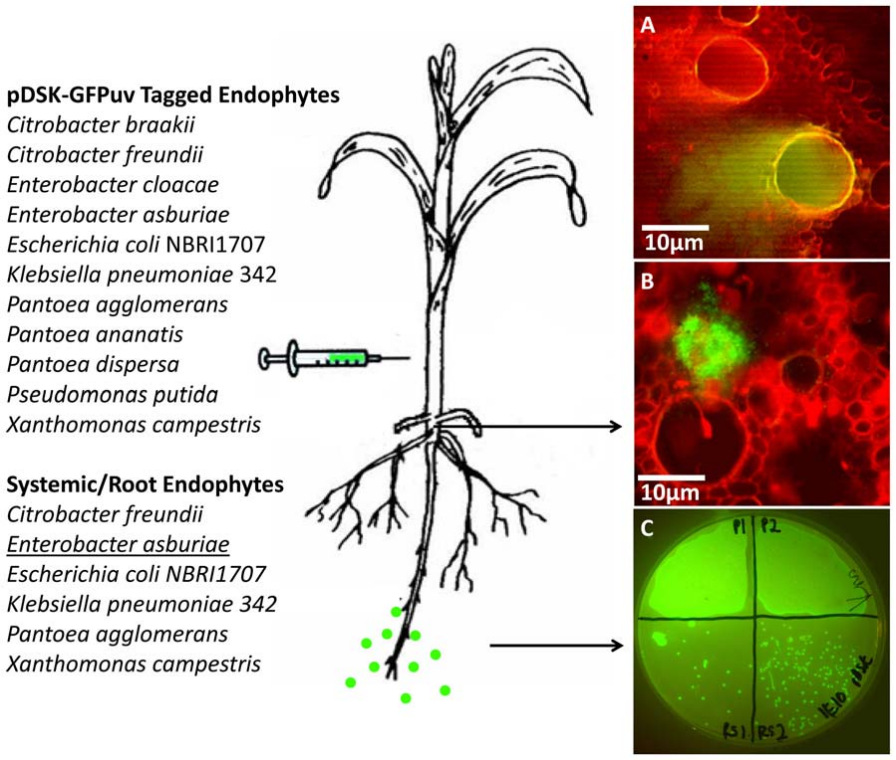
Persistence and migration of *Zea* seed endophytes in stems, roots and the rhizosphere. The 11 endophytes indicated were successfully tagged with GFP (pDSK-GFPuv, Kan^R^) (out of 124 isolates attempted) and injected into maize stems. The 6 endophytes indicated migrated to roots and persisted for >5 days as shown by fluorescence microscopy and culturing from macerated root tissues onto R2A Kanamycin media. (A) *Panteoa agglomerans* shown spilling out of a metaxylem vessel. (B) *Enterobacter asburiae* spilling out of root vascular tissue. (C) Culturing confirmed that *E. asburiae* was present in the roots of two plants (top two quandrants) as well as in their rhizospheres (bottom two quadrants).

A number of seed endophytes had functions that suggested they might be important in the roots and rhizosphere including phosphate solubilization. To track seed endophytes in the maize plant body, 11 species of endophytes that had been successfully transformed with a broad host range constitutive GFP expressing vector pDSK-GFPuv [45] were injected into the stems of Pioneer 3751 plants (Figure 10). After 5 days of growth, plant roots were sampled for microbes using microscopy and culturing on selective agar media. *Panteoa agglomerans* isolated from B73 was observed in metaxylem vessels (Figure 10A) and *Enterobacter asburiae* isolated from Diploperennis in phloem cells at the base of plant stems (Figure 10B) demonstrating their ability to move systemically through vascular tissues. The plate recovery method also showed that these and several additional microbes could migrate to roots, including *Citrobacter freundii* from Nicaraguensis, *Klebsiella pneumoniae 342* from Nicaraguensis, *E. coli NBRI1707* from Chapalote, and *Xanthomonas campestris* from B73 (Figure 10C). These results also confirm that these microbes were endophytes.

To evaluate the ability of the endophytes to exit the plant and colonize the surrounding rhizosphere, injections were made into stems of plants growing on agar in test tubes, and rhizosphere rinses were taken for plating. Interestingly, two isolates of *E. asburiae* were observed to colonize the rhizosphere from inside the plant (Figure 10C). This result suggests that in addition to chemical exudates that plants may secrete into the surrounding soil, they may also be able to directly release endophytes into the soil to amend it microbially.

### Phylogenetic analysis shows that the majority of *Zea* seed endophytes are γ-proteobacteria

**Figure 11:**
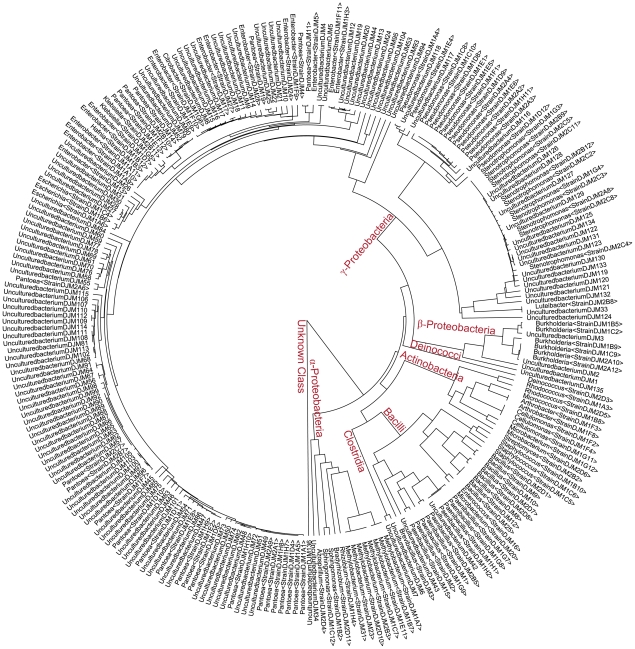
A phylogenetic tree of bacterial 16S rDNA sequences from *Zea* seed endophyte clones and cultured isolates. A multisequence alignment of the 16S region bounded by basepairs 867–1458 on an *E. coli* K12 reference sequence was used to generate a UPGMA tree. Included are sequences from clones (UnculturedbacteriumDJMX) and cultured isolates (Genus<StrainDJM-Plate#>) which are identified in Tables S2 and S3. Bacterial classes are labelled in red letters at major branch points.

Finally, in order to gain an understanding of the phylogenetic relationships between endophytes isolated from *Zea* seeds, 16S rDNA sequences from all clones and cultured isolates were aligned and trimmed to a region pertaining to base pairs 867–1458 on an *E. coli* K12 reference 16S sequence, and this alignment was then used to construct a UPGMA tree (Figure 11). This phylogenetic analysis shows γ-proteobacteria were the most abundant class of microbes observed in this study, with *Enterobacter* and *Panteoa* as the most common genera. Also represented were the classes α-proteobacteria, β-proteobacteria, Bacilli, Actinobacteria, Clostridia, Deinococci and an unknown class. Clostridia and the unknown class were only represented in the clone library. Conversely, the classes Actinobacteria and Deinococci were only represented by cultured isolates, with no cloned sequences observed.

## Discussion

This study was an attempt to understand the ecology of *Zea* seed endophytes. We used culture dependent and culture independent approaches to gain a complex picture of bacterial diversity and conservation amongst diverse *Zea* seeds and environments. Using TRFLP, we found *Zea* genotype-specific endophytes (Figure 3) and observed that seed endophyte diversity reflects the phylogenetic relationships of its *Zea* hosts (Figure 2A, 2B). We also observed conservation of seed endophytes in *Zea* across boundaries of evolution, ethnography and ecology (Figure 3A, 3B). Interestingly, endophytes in the wild ancestor persist today in domesticated maize (Figure 3A, 3B), though loss of the fruitcase during crop domestication may have altered the abundance of specific seed endophytes (Figure 3A, 3B). Many *Zea* seed endophytes solubilize phosphate, secrete acetoin and may fix nitrogen (Figure 8G) and a subset that were GFP tagged could be observed to migrate systemically to the root (Figure 10) and even rhizosphere (Figure 10). We found that endophyte communities in stem tissues are distinct from seeds but are highly similar across *Zea* genotypes (Figure 2C, 4).

### Conservation of microbial genera across *Zea* seed varieties

Maize was domesticated in southern Mexico from wild teosinte grasses [10] and subsequently became absolutely dependent on humans to propagate [46]. Despite 9,000 years of divergent selection and breeding by indigenous peoples and modern breeders, we observed that maize seeds have maintained a shared set of associated bacteria with their wild ancestors and with one another. TRFLP analysis of seeds (Figure 3A, 3B, 6) suggests that at least 4 bacterial groups including *Clostridium* spp. and *Paenibacillus* spp. are conserved across *Zea* groups despite differences in their genetics, geographic origin, and human use (Table 1). Our data suggests that the conserved bacteria are vertically transmitted between seed generations even following cross-continental migration (Figure 1). Our data does not exclude the possibility that other lesser abundant microbes may also be conserved [e.g. *Burkholderia/Herbaspirillum* (726 bp), Figure 5J], as TRFLP PCR only amplifies abundant targets (>1% of the sample) [47]. For example, *Pantoea* and *Enterobacter* ssp. appeared to be absent from nearly all maize seed based on TRFLP, but were in fact cultured from all groups of *Zea* seed (Figure 7). Metagenomic studies refer to conserved bacterial groups as the core microbiota of an organism, associated with healthy host functioning [48]. Conserved, vertically transmitted endophytes suggest an evolved form of mutualism or benign parasitism with their host plants [49]. Previous studies in other plants have also suggested the existence of core plant-associated microbiota [50], [51]. Endophytic diversity has been reported in maize and teosinte [14]–[33] but to our knowledge, this is the first report to suggest the existence of a core microbiota in *Zea* seeds.

### Host phylogenetic relationships determine the relatedness of resident bacterial communities

A major finding of this study is that *Zea* seed associated bacterial communities vary in accordance to host phylogeny (Figure 2A, 2B) similar to what has been shown in mammals by analysis of their microbial gut communities [34], [35]. During domestication, phylogenetic change in *Zea* involved selection against the fruitcase and glume tissues that protected ancestral seeds [41] which our results suggest may have altered endophyte titers (Figure 3A, 3B, 7). Based on a previous study [52], domestication was suggested to have altered seed-pathogen relationships: *Ustilago maydis*, an edible, obligate fungal pathogen of maize seeds, was shown to have undergone a dramatic genetic bottleneck 9,000 years ago at the time of maize domestication. In this study, we found no evidence for a major selection sweep during domestication with respect to seed endophyte community composition (Figure 7). Instead, humans appear to have gradually altered seed associated microbial communities perhaps by altering the seed habitat: ancestral maize seeds were small and hard in comparison to the diverse, large, starchy kernels [53] used today in a variety of indigenous foods [54]–[56] (Figure 1). Today, subsistence maize farmers in Mexico are known to select planting materials based on seed size and health rather than plant traits [57]. By choosing the largest, healthiest seeds, indigenous farmers may have selected against associated pathogens [58] and may have inadvertently caused shifts in seed endophyte populations. Modern breeding may have similarly shifted plant-associated microbial populations. For example, modern maize cultivars have been selected for increased benzoxazinoid (BX) production to combat insects and microbes, which is inferred to have altered fungal endophyte populations of *Fusarium* that are resistant to fungicidal BX byproducts [59]. With respect to the impact of modern breeding on pathogens, introgression of the plant male sterility allele *cms-t* into 85% of US maize acreage in the 1950s and 1960s, resulted in infections by a new strain of the pathogen *Bipolaris maydis* (race T), causing the Southern Corn Blight with ∼$1 billion in associated losses during the 1970's [60].

Given that host genotype is the critical factor regulating seed endophyte communities, it suggests developing *Zea* seeds are sheltered from infection by environmental microbes. *Zea* seeds develop within a maternally derived seed coat, which is further surounded by maternally derived tissues, including the fruitcase and glumes of teosinte, and the cob husk leaves of maize. It may be that specialized microbes colonizing these maternal tissue surfaces infect developing seeds similar to what has been observed in mammals: mice [61] and human fetuses [62] that initially develop in microbe-free wombs are colonized by differently composed microbe gut communities when they pass through the maternal vaginal canal compared to babies born by Caesarian section. In plants, it is also possible that bacterial seed endophytes are transmitted through direct vascular connections from the maternal parent, similar to the bacterial pathogen, *Pantoea stewartii*, which systemically spreads in maize from the shoot vasculature through the chalazal and into the seed endosperm [63]. Vertical transmission of bacteria would also be possible by colonization of shoot meristems. For example, the fungal endophytes *Neotyphodium* and *Epichloë* have been shown to be vertically transmitted through grass seed by initially infecting shoot apical meristems, which later become reproductive meristems; the endophytes persist as these cells give rise to ovules and seed [4], [64]. *Methylobacteria*, prominent in this study, have also been shown to intracellularly colonize pine meristems [65]. Finally, endophytes might be transferred through gametes directly: *Enterobacter cloacae* has been shown to be an endophyte of pollen grains of several species of Mediterranean pine [66]. Any of these mechanisms may explain the strong effect of host phylogeny on seed endophyte populations observed in this study.

Selection for plant body traits may also have altered endophytes found in seeds, the latter acting as vectors for vertical transmission of vegetative tissue endophytes. For example, one study showed it was possible to breed maize for improved microbial nitrogen fixation in roots [67]. We did observe that a significant percentage of endophytes are shared by both stems and seeds (Figure 3, 4, 6), and that some seed microbes can migrate to roots (Figure 10). However, we did not observe a phylogenetic∶endophyte correlation in stem tissues (Figure 2C). This may suggest stem endophytes of *Zea* do not have phenotypic effects on that tissue and thus have not have been targets in host evolutionary selection. Important caveats should be mentioned: as only the basal section of stems was sampled from each plant, it may be difficult to extrapolate our results to the whole shoot. Bacterial titers may also vary stochastically between host shoots, and even if there is small but important differences in endophytic populations, the TRFLP assay is biased for dominant microbial groups (Figure 4).

### Microbial ecology of *Zea* seed endophytes

We had hypothesized that there would be trait differences between microbes isolated from different *Zea* species based on the ecological niche of each host. However, across diverse *Zea* genotypes, the culturable endophyte community expressed a diverse range of phenotypes which were largely shared, including phosphate solubilization, growth on nitrogen-free media, and ability to produce acetoin or butanediol, which may serve as a strong growth promotion signal to the host plant [68] (Figure 8G). Since plants are composed of cellulose and pectin, it was not surprising to see that ∼30% of isolates were able to secrete cellulose and pectinase (Figure 8G). Cellulase is believed to be important in the endophytic colonization process by some types of bacteria [69]. As a cautionary note, similar to a previous study [14], it was disappointing, but not surprising that many of the bacteria observed by TRFLP (167 peaks) were not represented in the culture collection (31 genera), which perhaps limited our ability to resolve functional differences between host-specific communities of microbes.

The conserved suite of endophyte traits observed may reflect common needs of *Zea* seeds and their spermosphere, the soil surrounding germinating seed [70]. Most seeds spend part of their life cycle in soil. Seed associated microbes can play a role in promoting or resisting decay of the seed and preparing the surrounding soil environment for germination [71]. As seeds imbibe water during germination, they begin to secrete chemical exudates which are used as signals and energy sources by microbes, which quickly colonize the spermosphere, rhizosphere, and emerging seedling, where they can again antagonize pathogens, mineralize nutrients from the soil, promote germination and growth by producing hormones [70]. This concept is illustrated by cardon cactus, which can grow on bare rock, but has reduced seedling growth in the absence of bacterial seed endophytes [72]. The cactus endopohytes have been shown to provide the majority of nutrients to seedlings by enzymatically degrading rock and solubilising phosphate for the plant [72].

By far the most abundant seed endophytes in this study belonged to the class γ-Proteobacteria, whereas the most abundant maize root endophytes in another study [14] were observed to be Actinobacteria (cultured) and α-Proteobacteria (cloned). The most abundant culturable bacteria belonged to the genus *Enterobacter* (Figure 7) which were observed to survive on nitrogen free media, solubilise phosphate, produce acetoin and included 4/6 auxin producers in the study (Figure 9). These functions suggest that *Enterobacter* may help maize roots develop and acquire important nutrients from the soil. In seeds, *Enterobacter cloaceae* has been shown to be a very competitive and fast growing spermosphere colonist, which helps protect developing seedlings from pathogens by quickly consuming seed exudates, blocking their use by other microbes [70].

*Enterobacter* spp. are very closely related phylogenetically to *Pantoea*, the latter being a commonly cultured and even more oftenly cloned genus of endophytes in this study (Figure 7). Both genera belong to the class γ-Proteobacteria, and have previously been shown to be commonly culturable endophytes [73]. In this study, though 50% of *Enterobacter* spp. were potential nitrogen fixers, only 1/17 *Pantoea* spp. exhibited this trait although more *Pantoea* had ACC deaminase activity (Figure 9). *Pantoea* spp. are usually considered as plant pathogens responsible for soft rot [74], [75] which interestingly can also cause human disease [76]. Conversely, other *Pantoea* spp. are commensal or beneficial endophytes [77] that may be important in protecting seeds from fungal infection [9].

The third most commonly isolated endophytes belonged to *Pseudomonas* of which a disproportionate number of isolates exhibited growth on nitrogen free media, had ACC deaminase activity, and produced RNase, cellulase and pectinase (Figure 9). However, *Pseudomonas* spp. showed low acetoin and no auxin production, suggesting they are not involved in manipulating *Zea* hormones. Previous studies have shown that *Pseudomonas* ssp. are common soil, rhizosphere and endosphere inhabitants, with roles ranging from soil disease suppression by chelation of available iron [78], root growth stimulation through ACC deaminase activity [79], and plant growth promotion [80]. *Pseudomonas* spp. have also been shown to be important plant pathogens which can disrupt other plant endophytes causing indirect effects on plant health [81].

*Methylobacteria*, easily identifiable as pink colonies, were also widely cultured from diverse maize genotypes (Figure 7) but were slow growers in our study. *Methylobacteria* are named for their phyllosophere habit of metabolizing volatile methanol emitted from stomata [82], [83] and may thus be able to modulate airborne signals emitted by plants. In previous studies, some *Methylobacteria* ssp. were shown to be xylem [84] and seed endophytes [7] and were able to fix nitrogen, antagonize pathogens, promote seedling germination and plant growth through ACC deaminase activity and hormone production [85], [86].

### Do endophytes contribute to unique *Zea* traits?

Of particular interest in this study was to functionally characterize endophytes found in wild *Zea* species (teosintes) in addition to two giant maize landraces, Mixteco and Jala. As teosintes grow without inputs provided by humans, we expected their endophytes to be enriched in nutrient acquisition and biocontrol functions. The endophytes of two of the teosintes, Diploperennis and Nicaraguensis, appeared to be over-represented for antibiosis activities, growth on nitrogen free media, and ability to produce siderophores and auxin, but this was less obvious in the remaining teosintes, Mexicana and Parviglumis (Figure 8G). Furthermore, the microbial titres of all 4 teosinte seeds were high relative to the titres observed in maize seed (Figure 3B, 7). The bleach/ethanol treatment used to surface sterilize maize seeds, was found to be insufficient to sterilize teosinte seeds (data not shown), suggesting that the teosinte fruitcase helps protect and house microbes.

Out of 91 microbial isolates tested, only 6 produced auxin. Four of the six auxin-producing endophytes were cultured from Nicaraguensis (*Stenotrophomonas maltophilia*, *Enterobacter asburiae*, and two isolates of *Enterobacter hormaechei*, Figure 8G). Nicaraguensis is a unique *Zea* genotype known to be highly flood tolerant as it inhabits seasonally flooded coastal plains and estuaries in its native Nicaragua [36]. Flood tolerance in Nicaraguensis is conferred by specialized root traits including the presence of aerenchyma for oxygen transport to roots [87] as well as the formation of adventitious roots which can grow in the air or at the soil surface [88]. As adventitious root formation is strongly induced by auxin [89] our results may reflect natural selection on Nicaraguensis for auxin producing endophytes. The fifth auxin producing endophyte was isolated from Diploperennis (*Enterobacter hormaechei*, Table S4). Diploperennis was a unique *Zea* genotype in this study as it is perennial [90]. A large, persistent root system is a well known adaptation for perennialism [90], and one possibility is that it was enhanced in Diploperennis by the auxin producing endophyte. The last auxin producing endophyte was cultured from the seeds of Jala (*Pantoae ananatis*), and bacteria with this trait that we found in maize (Figure 8G). Jala is known locally in the Mexican state of Jalisco as “maiz de humedo” (moist-soil maize) [38], and thus may also benefit from root growth promoting endophytes. As noted above, we were particularly interested in Jala because it is a giant corn plant; in fact it may be the world's tallest maize, growing as high as 6 m, the grain harvested by horseback [38]. A plant with a large shoot would benefit from an auxin producing endophyte by promoting root growth for enhanced anchorage and nutrient acquisition.

Of major interest in this study was the ability of isolates to promote plant growth. The only *Zea* seed endophyte that reproducibly promoted shoot growth in a potato bioassay was isolated from Mixteco (*Burkholderia phytofirmans*) (Figure 8G). Mixteco was the other giant maize in this study. *B. phytofirmans* is a known plant growth promoting endophyte [91] which has been fully sequenced and until now was only ever isolated from sphagnum moss, onion, and rice [92]. Although many isolates displayed potentially beneficial traits *in vitro*, most of the other isolates tested appeared to stunt potato root and shoot biomass (Figure 9). This may not reflect the behaviour of these endophytes inside *Zea* seed or plants, as it has been previously observed that endophytes confer growth promotion in a host-specific manner [93]. *Zea* and potato are also very different genetically, being separated by >100 million years of evolution [94]. For example, from Nicaraguensis seed we isolated a strain with 100% identity to *Klebsiella pneumoniae* 342, a growth promoter of corn under field conditions [95], but it caused growth inhibition of gnotobiotic potatoes. A gnotobiotic corn growth promotion assay does not yet exist, but would be useful for future experiments in this area.

### *Zea* seed endophytes can colonize the roots and rhizosphere

We tagged seed associated microbes with GFP and injected them into shoots in order to confirm their endophyte behavior. Several of the microbes were able to persist and systemically travel to the roots, confirming that they are endophytes (Figure 10). Of particular interest was *Enterobacter asburaie*, a previously reported endophyte [96], [97] which was also able to exit the plant and colonize the rhizosphere (Figure 10C). We observed that *E. asburiae* has cellulase activity (Table S4), and a previous study reported that it had the ability to bore holes in cotton to facilitate endophytic colonization [69] suggestive of a mechanism for how it might exit roots. *E. asburiae* was the strongest of the auxin producers isolated from Nicaraguensis (Table S4), consistent with a previous study showing it to secrete auxin in cowpea [98]. *E. asburiae* was also able to grown on nitrogen free media (Table S4). Similar to other seed associated microbes isolated in this study, *E. asburiae* was able to solubilize phosphate (Table S4), a trait that would only be beneficial to the plant if the microbe could inhabit the rhizosphere. Though phosphate solubilization can be conferred by weak acid production, Gyaneshwar *et al.* [99] found that the only isolates that had this ability under highly buffered conditions in the rhizosphere of pigeon pea were *E. asburiae*. These results show that some seed associated microbes are competent to colonize the vegetative organs of the plant and may even be able to exit the plant and colonize the rhizosphere.

In conclusion, it appears that *Zea* has a core microbiota that is conserved across maize evolution, domestication and migration. However, this study has also demonstrated that seeds are a good source for discovering host genotype-specific endophytes. All of these endophytes displayed a range of functions *in vitro*, and future *in planta* studies will be needed to determine how they contribute to the life cycles of their hosts.

## Methods

### Sources of first generation seeds

The immediate parents of the seeds came from different geographic locations (Table 1). Except if noted, all seeds were obtained from the International Maize and Wheat Improvement Center (CIMMYT) (Texcoco, Mexico) and accession numbers are provided (Table 1). Pioneer 3751 seed were treated with both MaximXL, a fungicide that controls *Pythium* and *Rhizoctonia* and ApronXL which controls *Pythium* and *Phytophthora*.

### Sources of first generation stems and second generation seeds

To investigate the effect of environment on *Zea* associated microbes, all genotypes were grown in a common garden. Ten plants per genotype were germinated in Petri dishes under wet paper towels, transferred individually to biodegradable, pressed cow manure pots, and filled with composted cow manure as soil. These were watered daily with tap water in a growth room maintained at 28°C with a 10 hour photoperiod to ensure that the Mexican varieties (short day plants) would flower in the field. Following 30 days, plants in pots were transplanted in a randomized plot design to a corn field near Guelph, Canada, at GPS coordinates: latitude, 43.49556918428844 and longitude -80.32565832138062. At the time of seed harvest in late fall, 10 cm long stem sections from all plants were taken from just above the top crown root. No stem samples were obtainable from Gaspe, nor Generation 2 seed from Jala, Mixteco, Nal-Tel, Tuxpeno or *Zea nicaraguensis*.

### Seed and stem surface sterilization

To soften seed and revive endophytic populations, 15 seeds per genotype were soaked in distilled water for 48 hours, drained, and seeds washed in 0.1% Triton X-100 detergent for 10 min with shaking. This water was drained, and seeds washed with 3% sodium hypochlorite for 10 min. The bleach was drained, and a 3% sodium hypochlorite wash repeated for an additional 10 min. The seeds were then drained and rinsed with autoclaved, distilled water, before being washed for 10 min in 95% ethanol for 10 min. The ethanol wash was drained, and seeds rinsed three times with autoclaved, distilled water. To check for surface sterility, 5 seeds per treatment were momentarily placed on sterile R2A agar plates and these plates incubated for 10 days at 25°C.

As stem sections were much larger than seeds, they were individually surface sterilized by immersion in 95% ethanol for 3 minutes, removed from the ethanol bath with forceps, and flamed. This step was repeated twice for each stem section.

### Bacterial extraction

In order to extract bacteria from surface sterilized samples, 15 seeds/genotype were ground in an autoclaved mortar and pestle to which was added 1 ml of 50 mM Na_2_HPO_4_ buffer per gram of seed dry weight (teosintes received 2 ml/g). 1 ml of this mixture was added to an Eppendorf tube and frozen for later DNA extraction; for culturing, 50 µl was serially diluted three times in 450 µl of 50 mM Na_2_HPO_4_ buffer, resulting in 10×, 100×, and 1000× dilutions.

Clean stems were ground in a flame-sterilized, metal Waring blender resulting in a woody pulp, to which 0.5 ml of 50 mM Na_2_HPO_4_ buffer was added per gram of tissue. 1 ml was frozen and used for DNA extraction.

### DNA extraction and Terminal Restriction Fragment Length Polymorphism (TRFLP)

Total DNA was extracted from 1 ml of extract using DNeasy Plant Mini Kits (Qiagen, USA), and eluted in water. DNA was quantified (Nanodrop, Thermo Scientific, USA). A PCR mastermix was made with the following components per 25 µl volume: 2.5 µl Standard Taq Buffer (New England Biolabs), 0.5 µl of 25 mM dNTP mix, 0.5 µl of 10 mM 27F-Degen primer with sequence AGRRTTYGATYMTGGYTYAG [100], 0.5 µl of 10 mM 1492r primer with sequence GGTTACCTTGTTACGACTT [100], 0.25 µl of 50 mM MgCl_2_, 0.25 µl of Standard Taq (New England Biolabs), 50 ng of total DNA, and double distilled water up to 25 µl total. Amplification was for 35 cycles in a PTC200 DNA Thermal Cycler (MJ Scientific, USA) using the following program: 96°C for 3 min, 35× (94°C for 30 sec, 48°C for 30 sec, 72°C for 1∶30 min), 72°C for 7 min.

Using the same conditions as above, 1.5 µl of the above PCR product was used as a template in a nested, fluorescently labelled PCR reaction. For the nested PCR, primer 799f with sequence AACMGGATTAGATACCCKG [14] was labelled with 6FAM, and 1492rh with sequence HGGHTACCTTGTTACGACTT was labelled with Max550, both by Integrated DNA Technologies (USA). 1.5 µl of the labelled PCR product was then added to 8.5 µl restriction mixture [1U *Dde*I (NEB), 1X Buffer 3 (NEB)] and incubated in darkness at 37°C for 16 hours before sequencing gel analysis using a 3730 DNA Analyzer alongside GeneScan 1200 LIZ Size Standards (Applied Biosystems, USA).

TRFLP amplification and restriction was repeated three times for all seed and stem samples.

### TRFLP analysis

TRFLP results were analyzed using Peak Scanner software (Applied Biosystems, USA) using default settings with a modified fragment peak height cut off of 35 fluorescence units. The forward and reverse fragment sizes plus peak heights were exported to Microsoft Excel. Primer dimers, chloroplast 16S rDNA and 18S rDNA fragments were removed (peaks 1–19, 40, 50, 51, 356, 357, 358, 362, 513, 515, 526 bp).

To generate pseudo TRFLP profiles for display, only forward fragements were used. The TRFLP fragment intensity for each peak was calculated for each PCR trial by subtracting the water control intensity; the results from all PCR trials were then averaged.

For PCA analysis, both forward and reverse fragments were used. The analysis used the frequency of PCR trials with which each peak was detected rather than peak intensity. PCA analysis of covariance was done using GGEbiplot software (Canada).

### Seed bacterial 16S rDNA clone library generation and sequencing

10 µl of each nested PCR product was run on an electrophoresis gel, and the 693 bp band was gel extracted and ligated to PCR cloning vector, pDRIVE (Qiagen, USA). 48 clones from each transformation were screened by colony PCR using plasmid primers M13 Forward and M13 Reverse, combined with amplified ribosomal DNA restriction analysis (ARDRA, [101]) with *Dde*I. Clones with distinct restriction patterns were subjected to colony PCR sequencing, and reads were BLAST searched against bacterial 16S in Genbank [102]. A total of 127 clone sequences (≥200 bp) were submitted to Genbank and received accession numbers JF753273 to JF753400; these are also identified in Table S2. To predict fragment sizes from clones and cultures, sequences were submitted to the *in silico* TRFLP analysis program TRiFLe [103].

### Endophyte culturing and 16S rDNA identification

50 µl of 10× and 1000× dilutions of each plant tissue extract was spread on three different types of agar media: LGI (50 g sucrose, 0.01 g FeCl_3_-6H_2_O, 0.8 g K_3_PO_4_, 0.2 g MgSO_4_-7H_2_0, 0.002 g Na_2_MoO_4_-2H_2_O, Agar 7.5 g/l, pH 7.5) for diazotrophic bacteria; R2A (#17209, Sigma) media for oligotrophic bacteria, and potato dextrose agar (PDA) (#70139, Sigma) for copiotrophic bacteria and fungi. To prevent fungal growth on R2A and LGI plates, 20 µl of 100 mM cyclohexamide was spread on the surface of plates before inoculation. Plates were incubated at 25°C for 10 days.

Unique bacteria from each plate were chosen based on colony colour and morphology. For identification, colony PCR was undertaken as above using primers 27f-Degen and 1492r; when a clean 1465 bp amplicon was present, 1 µl was used directly as template in a sequencing reaction. The sequencing reaction used primer 787f (AATAGATACCCNGGTAG), with an annealing temperature of 49°C, and standard BigDye reaction conditions (Applied Biosystems, USA). If necessary, amplicons were gel purified before sequencing. Reads were BLAST searched against bacterial 16S Genbank [102] and 152≥200 bp were deposited in Genbank with accession numbers JF753401 to JF753552; these are also identified in Table S3.

For phylogenetic comparison of clone and culture 16S sequences, all reads were aligned using CLUSTALW, trimmed using Bioedit software [104] to a mutually overlapping region pertaining to basepairs 867–1458 on an *E. coli* K12 reference 16S rDNA sequence, and UPGMA phylogenetic trees generated and visualized using Geneious software [105].

### Bacterial phenotyping

Each endophyte was subjected to thirteen phenotypic tests using 96 well replica plating when possible. All tests were performed in duplicate, except the plant growth promotion assay which was carried out in triplicate. The different protocols used are described as follows:

#### Plant growth promotion assay

We assayed for growth effects of each bacteria on nodal explants of potato (*Solanum tuberosum* cv. Kennebec) [43]. For potato growth, potato nodal cutting media (PNCM) was prepared [4.4 g/L of MS Basal Salts with Minimal Organics (#M6899, Sigma), 15 g/L of sucrose, 7.5 g/L of agar, and a pH of 6] and 10 mL poured into 22×150 mm tissue culture tubes. Bacterial colonies were resuspended in 50 mM Na_2_HPO_4_ (pH 7) to an OD_600_ of 0.2, and 100 µl injected onto each plantlet. Triplicate tubes were incubated for 30 days in a growth chamber with 50% humidity, 16 hour photoperiod (100 µM m−2 sec−1 with incandescent and fluorescent lights), with 24°C day and 16°C night. After 30 days, agar was removed from roots, and fresh weights of roots and shoots recorded compared to the mean of 9 buffer treated plantlets.

#### Growth on nitrogen free LGI media

All glassware was cleaned with 6 M HCl before media preparation. A new 96 deep-well plate (2 ml well volume) was filled with 1 ml/well of sterile LGI broth [per L, 50 g Sucrose, 0.01 g FeCl_3_-6H_2_O, 0.8 g K_3_PO_4_, 0.2 g MgSO_4_-7H_2_O, 0.002 g Na_2_MoO_4_-2H_2_O, pH 7.5]. Bacteria were inoculated with a flame-sterilized 96 pin replicator. The plate was sealed with a breathable membrane, incubated at 25°C with gentle shaking for 5 days, and OD_600_ readings taken.

#### ACC Deaminase Activity

Microbes were assayed for growth with ACC as their sole source of nitrogen. Prior to media preparation all glassware was cleaned with 6 M HCl. A 2 M filter sterilized solution of ACC (#1373A, Research Organics, USA) was prepared in water. 1 µl/mL of this was added to autoclaved LGI broth (see above), and 1 mL aliquots were placed in a new 96 well plate. The plate was sealed with a breathable membrane, incubated at 25°C with gentle shaking for 5 days, and OD600 readings taken. Only wells that were significantly more turbid than their corresponding nitrogen free LGI wells were considered to display ACC deaminase activity.

#### Mineral Phosphate Solubilization

Microbes were plated on tricalcium phosphate media [106]. This was prepared as follows: 10 g/L glucose, 0.373 g/L NH_4_NO_3_, 0.41 g/L MgSO_4_, 0.295 g/L NaCl, 0.003 FeCl_3_, 0.7 g/L Ca_3_HPO_4_ and 20 g/L Agar, pH 6, then autoclaved and poured into 150 mm plates. After 3 days of growth at 25°C in darkness, clear halos were measured around colonies able to solubilize the tricalcium phosphate.

#### RNAse activity

1.5 g of torula yeast RNA (#R6625, Sigma) [107] was dissolved in 1 mL of 0.1 M Na_2_HPO_4_ at pH 8, filter sterilized and added to 250 mL of autoclaved R2A agar media which was poured into 150 mm plates. The bacterial endophytes from a glycerol stock plate were inoculated using a flame-sterilized 96 pin replicator, and incubated at 25°C for 3 days. On day three, plates were flooded with 70% perchloric acid (#311421, Sigma) for 15 minutes and scored for clear halo production around colonies.

#### Acetoin and diacetyl production

The method was adapted [108].1 ml of autoclaved R2A broth supplemented with 0.5% glucose was aliquoted into a 96 deep well plate (#07-200-700, Fisher). The bacterial endophytes from a glycerol stock plate were inoculated using a flame-sterilized 96 pin replicator, sealed with a breathable membrane, then incubated for 5 days with shaking (200 rpm) at 25°C. At day 5, 100 µl aliquots of culture were removed and placed into a 96 well white fluorometer plate, along with 100 µl/well of Barritt's Reagents A and B which were prepared by mixing 5 g/L creatine mixed 3∶1 (v/v) with freshly prepared ∝-naphthol (75 g/L in 2.5 M sodium hydroxide). After 15 minutes, plates were scored for red or pink colouration against a copper coloured negative control.

#### Auxin production

R2A agar media, supplemented with L-tryptophan to a final concentration of 5 mM, was autoclaved and poured into 150 mm plates [109]. Using a 96 pin plate replicator, all seed endophytes were inoculated onto the fresh plate from a 96 well plate glycerol stock. The plate was incubated at 25°C for 3 days, then overlaid with a nitrocellulose membrane, and put in a fridge at 4°C overnight, allowing bacteria and their metabolites to infiltrate into the paper. The next day, the nitrocellulose membrane was removed and placed for 30 min on Whatman #2 filter papers saturated with Salkowski reagent (0.01 M ferric chloride in 35% perchloric acid, #311421, Sigma). Dark pink halos around colonies were visualized in the membrane by background illumination using a light table.

#### Siderophore production

To ensure no contaminating iron was carried over from previous experiments, all glassware was deferrated with 6 M HCl and water prior to media preparation [110]. In this cleaned glassware, R2A agar media, which is iron limited, was prepared and poured into 150 mm Petri dishes and inoculated with bacteria using a 96 pin plate replicator. After 3 days of incubation at 25°C, plates were overlaid with O-CAS overlay [111]. Again using the cleaned glassware, 1 liter of O-CAS overlay was made by mixing 60.5 mg of Chrome azurol S (CAS), 72.9 mg of hexadecyltrimethyl ammonium bromide (HDTMA), 30.24 g of finely crushed Piperazine-1,4-bis-2-ethanesulfonic acid (PIPES) with 10 ml of 1 mM FeCl_3_·6H_2_O in 10 mM HCl solvent. The PIPES had to be finely powdered and mixed gently with stirring (not shaking) to avoid producing bubbles, until a dark blue colour was achieved. Melted 1% agarose was then added to pre-warmed O-CAS just prior pouring the overlay in a proportion of 1∶3 (v/v). After 15 minutes, colour change was scored by looking for purple halos (catechol type siderophores) or orange colonies (hydroxamate siderophores).

#### Pectinase activity

Adapting a previous protocol [112] 0.2%(w/v) of citrus pectin (#76280, Sigma) and 0.1% triton X-100 were added to R2A media, autoclaved and poured into 150 mm plates. Bacteria were inoculated using a 96 pin plate replicator. After 3 days of culturing in the darkness at 25°C, pectinase activity was visualized by flooding the plate with Gram's iodine. Positive colonies were surrounded by clear halos.

#### Cellulase activity

Adapting a previous protocol [113], 0.2% carboxymethylcellulose (CMC) sodium salt (#C5678, Sigma) and 0.1% triton X-100 were added to R2A media, autoclaved and poured into 150 mm plates. Bacteria were inoculated using a 96 pin plate replicator. After 3 days of culturing in the darkness at 25°C, cellulose activity was visualized by flooding the plate with Gram's iodine. Positive colonies were surrounded by clear halos.

#### Antibiosis

Bacteria were inoculated using a 96 pin plate replicator onto 150 mm Petri dishes containing R2A agar, then grown for 3 days at 25°C. At this time, colonies of either *E. coli* DH5α (gram negative tester), *Bacillus subtillus* ssp. *Subtilis* (gram positive tester), or yeast strain AH109 (fungal tester) were resuspended in 1 mL of 50 mM Na_2_HPO_4_ buffer to an OD_600_ of 0.2, and 30 µl of this was mixed with 30 mL of warm LB agar. This was quickly poured completely over an endophyte array plate, allowed to solidify and incubated at 37°C for 16 hours. Antibiosis was scored by looking for clear halos around endophyte colonies.

### GFP tagging, plant inoculations and microscopy

Broad scale GFP tagging was attempted with wide-host vector pDSK-GFPuv [45]. Electrocompetent endophytes were prepared by repeated pelleting and washing with cold, distilled water and electroporated using standard procedures except SOC was substituted with R2A broth; strains displaying poor growth were allowed to grow up to 5 days or until cultures appeared to have reached an OD_600_ of 0.5–1.0. For electroporation, 1 ml of cold R2A (plus 0.5% glucose) media instead of LB/SOC was added to the cuvette following electroporation; cultures were shaken at 37°C for 2 hours before plating onto R2A agar with 50 µg/ml Kanamycin.

To verify endophyte habit and ability to migrate to roots, GFP-tagged endophyte cultures were resuspended in 50 mM Na_2_HPO_4_ buffer (OD_600_ of 0.2), and 20 µl was inoculated into stems of 5-leaf-tip staged maize plants (Pioneer 3751) 10 cm about the soil. Plants were grown in Turface clay media with Hoagland's solution. For each inoculation, the tip of an Exacto knife was inserted into the stem, removed, and then each culture was injected using a standard 20 µl pipette tip. Five days later, roots of injected plants were macerated in sterile mortars and pestles, mixed with 10 ml of 50 mM Na_2_HPO_4_ buffer, and 100 µl spread onto R2A agar containing 50 µg/ml Kanamycin for GFP visualization. This test was repeated twice for all transformed endophytes.

For microscope analysis, and to test the ability of endophytes to colonize the rhizosphere from inside the plant, endophyte injection was repeated using 3-leaf-tip stage maize seedlings (Pioneer 3751) growing in glass tubes (22×150 mm) containing 20 ml of sucrose-free PNCM agar (see above). Inoculations were as above except 10 µl of each endophyte culture (OD_600_ = 0.2) was used. Five days later, seedlings were removed. To each tube, 2 ml of 50 mM Na_2_HPO_4_ buffer was added to the remaining agar, swirled and then decanted onto R2A agar containing 50 µg/ml Kanamycin. Roots were again macerated as above and plated onto R2A agar containing 50 µg/ml Kanamycin.

Hand sections of the root region just below crown root emergence zone and above the hilum were taken, stained with 5 mM propidium iodide (Sigma) and screened for GFP expression using a Leica fluorescent microscope and Northern Eclipse software.

## Supporting Information

Table S1 **TRFLP fragments sizes and intensities from three repetitions done on stems, Generation 1 and 2 seed.**
(XLSX)

Table S2 **Bacterial 16S rDNA sequences amplified and cloned from seed DNA.**
(XLSX)

Table S3 **Bacterial 16S rDNA sequences from pure cultures of bacteria isolated from Generation 1 and Generation 2 seed.**
(XLSX)

Table S4 **Detailed information of** ***in vitro*** **functional traits observed in cultured seed endophytes.**
(XLSX)

## References

[1] Johnston-Monje D, Raizada MN, Moo-Young M (2011). Plant and endophyte relationships: Nutrient management.. Comprehensive Biotechnology. 2 ed.

[2] Mundt JO, Hinkle NF (1976). Bacteria within ovules and seeds.. Appl Environ Microbiol.

[3] Kremer RJ (1987). Identity and properties of bacteria inhabiting seeds of selected broadleaf weed species.. Microb Ecol.

[4] Schardl CL, Leuchtmann A, Spiering MJ (2004). Symbioses of grasses with seedborne fungal endophytes.. Annu Rev Plant Biol.

[5] Dalling JW, Davis AS, Schutte BJ, Arnold AE (2011). Seed survival in soil: interacting effects of predation, dormancy and the soil microbial community.. J Ecol.

[6] Kaga H, Mano H, Tanaka F, Watanabe A, Kaneko S (2009). Rice seeds as sources of endophytic bacteria.. Microb Environ.

[7] Mano H, Tanaka F, Watanabe A, Kaga H, Okunishi S (2006). Culturable surface and endophytic bacterial flora of the maturing seeds of rice plants (*Oryza sativa*) cultivated in a paddy field.. Microb Environ.

[8] Ferreira A, Quecine MC, Lacava PT, Oda S, Azevedo JL (2008). Diversity of endophytic bacteria from Eucalyptus species seeds and colonization of seedlings by *Pantoea agglomerans*.. FEMS Microbiol Lett.

[9] Rijavec T, Lapanje A, Dermastia M, Rupnik M (2007). Isolation of bacterial endophytes from germinated maize kernels.. Can J Microbiol.

[10] Matsuoka Y, Vigouroux Y, Goodman MM, Sanchez GJ, Buckler E (2002). A single domestication for maize shown by multilocus microsatellite genotyping.. Proc Natl Acad Sci U S A.

[11] Wilkes G, Smith CW, Betran J, Runge ECA (2004). Corn, strange and marvelous: But is a definitive origin known?. Corn: Origin, History, Technology, and Production.

[12] Turrent A, Serratos JA, Sarukhán J, Raven P (2004). Context and background on maize and its wild relatives in Mexico.. Maize and biodiversity: The effects of transgenic maize in Mexico.

[13] Ruiz Corral JA, Durán Puga N, Sánchez González JdJ, Ron Parra J, González Eguiarte DR (2008). Climatic adaptation and ecological descriptors of 42 Mexican maize races.. Crop Sci.

[14] Chelius MK, Triplett EW (2001). The diversity of archaea and bacteria in association with the roots of *Zea mays* L.. Microb Ecol.

[15] McInroy JA, Kloepper JW (1995). Survey of indigenous bacterial endophytes from cotton and sweet corn.. Plant Soil.

[16] Estrada P, Mavingui P, Cournoyer B, Fontaine F, Balandreau J (2002). A N2-fixing endophytic *Burkholderia* sp. associated with maize plants cultivated in Mexico.. Can J Microbiol.

[17] Roesch L, Camargo F, Bento F, Triplett E (2007). Biodiversity of diazotrophic bacteria within the soil, root and stem of field-grown maize.. Plant Soil.

[18] Reis VM, Estrada-de los Santos P, Tenorio-Salgado S, Vogel J, Stoffels M (2004). *Burkholderia tropica* sp. nov., a novel nitrogen-fixing, plant-associated bacterium.. Int J Syst Evol Microbiol.

[19] Caballero-Mellado J, Martinez-Aguilar L, Paredes-Valdez G, Santos PE (2004). *Burkholderia unamae* sp. nov., an N2-fixing rhizospheric and endophytic species.. Int J Syst Evol Microbiol.

[20] Seghers D, Wittebolle L, Top EM, Verstraete W, Siciliano SD (2004). Impact of agricultural practices on the *Zea mays* L. endophytic community.. Appl Environ Microbiol.

[21] Nassar AH, El-Tarabily KA, Sivasithamparam K (2005). Promotion of plant growth by an auxin-producing isolate of the yeast *Williopsis saturnus* endophytic in maize (*Zea mays* L.) roots.. Biol Fertil Soils.

[22] Palus JA, Borneman J, Ludden PW, Triplett EW (1996). A diazotrophic bacterial endophyte isolated from stems of *Zea mays* L. and *Zea luxurians* Iltis and Doebley.. Plant Soil.

[23] Hinton DM, Bacon CW (1995). *Enterobacter cloacae* is an endophytic symbiont of corn.. Mycopathologia.

[24] Zinniel DK, Lambrecht P, Harris NB, Feng Z, Kuczmarski D (2002). Isolation and characterization of endophytic colonizing bacteria from agronomic crops and prairie plants.. Appl Environ Microbiol.

[25] Chelius MK, Henn JA, Triplett EW (2002). *Runella zeae* sp. nov., a novel Gram-negative bacterium from the stems of surface-sterilized *Zea mays*.. Int J Syst Evol Microbiol.

[26] Rosenblueth M, Martínez-Romero E (2004). *Rhizobium etli* maize populations and their competitiveness for root colonization.. Arch Microbiol.

[27] Montañez A, Abreu C, Gill P, Hardarson G, Sicardi M (2009). Biological nitrogen fixation in maize (*Zea mays* L.) by 15N isotope-dilution and identification of associated culturable diazotrophs.. Biol Fertil Soils.

[28] Figueiredo JEF, Gomes EA, Guimarães CT, Lana UGP, Teixeira MA (2009). Molecular analysis of endophytic bacteria from the genus *Bacillus* isolated from tropical maize (*Zea mays* L.).. Braz J Microbiol.

[29] Ersayin Yasinok A, Şahin FI, Haberal M (2008). Isolation of endopyhtic and xylanolytic *Bacillus pumilus* strains from *Zea mays*.. Tarim Blmler Dergs.

[30] Roesch LFW, Passaglia LMP, Bento FM, Triplett EW, Camargo FAO (2007). Diversity of diazotrophic endophytic bacteria associated with maize plants.. Rev Bras Ciência Solo.

[31] McInroy JA, Kloepper JW (1995). Population dynamics of endophytic bacteria in field-grown sweet corn and cotton.. Can J Microbiol.

[32] Fisher PJ, Petrini O, Scott HML (1992). The distribution of some fungal and bacterial endophytes in maize (*Zea mays* L.).. New Phytol.

[33] Rai R, Dash PK, Prasanna BM, Singh A (2007). Endophytic bacterial flora in the stem tissue of a tropical maize (*Zea mays* L.) genotype: isolation, identification and enumeration.. World J Microbiol Biotechnol.

[34] Ley RE, Hamady M, Lozupone C, Turnbaugh PJ, Ramey RR (2008). Evolution of mammals and their gut microbes.. Science.

[35] Ley RE, Lozupone CA, Hamady M, Knight R, Gordon JI (2008). Worlds within worlds: evolution of the vertebrate gut microbiota.. Nat Rev Microbiol.

[36] Iltis HH, Benz BF (2000). *Zea nicaraguensis* (Poaceae), a new teosinte from pacific coastal Nicaragua.. Novon.

[37] Dalton DA, Kramer S, Gnanamanickam SS (2006). Nitrogen fixing bacteria in nonlegumes.. Plant-Associated Bacteria.

[38] Rice E (2007). Conservation in a changing world: *in situ* conservation of the giant maize of Jala.. Genet Resour Crop Evol.

[39] Schnable PS, Ware D, Fulton RS, Stein JC, Wei F (2009). The B73 maize genome: Complexity, diversity, and dynamics.. Science.

[40] Shyu C, Soule T, Bent SJ, Foster JA, Forney LJ (2007). MiCA: a web-based tool for the analysis of microbial communities based on terminal-restriction fragment length polymorphisms of 16S and 18S rRNA genes.. Microb Ecol.

[41] Wang H, Nussbaum-Wagler T, Li B, Zhao Q, Vigouroux Y (2005). The origin of the naked grains of maize.. Nature.

[42] Krakat N, Westphal A, Schmidt S, Scherer P (2010). Anaerobic digestion of renewable biomass: Thermophilic temperature governs methanogen population dynamics.. Appl Environ Microbiol.

[43] Conn KL, Lazarovits G, Nowak J (1997). A gnotobiotic bioassay for studying interactions between potatoes and plant growth-promoting rhizobacteria.. Can J Microbiol.

[44] Ågren GI, Franklin O (2003). Root∶shoot ratios, optimization and nitrogen productivity.. Ann Bot (Lond).

[45] Wang K, Kang L, Anand A, Lazarovits G, Mysore KS (2007). Monitoring in planta bacterial infection at both cellular and whole-plant levels using the green fluorescent protein variant *GFPuv*.. New Phytol.

[46] Vollbrecht E, Sigmon B (2005). Amazing grass: developmental genetics of maize domestication.. Biochem Soc Trans.

[47] Smalla K, Bull A (2004). Culture-independant microbiolgy.. Microbial Biodiversity and Bioprospecting.

[48] Sekelja M, Berget I, Nas T, Rudi K (2010). Unveiling an abundant core microbiota in the human adult colon by a phylogroup-independent searching approach.. ISME J.

[49] Ewald PW (1987). Transmission modes and evolution of the parasitism-mutualism continuuma.. Ann N Y Acad Sci.

[50] Inceoglu O, Salles JF, van Overbeek L, van Elsas JD (2010). Effects of plant genotype and growth stage on the betaproteobacterial communities associated with different potato cultivars in two fields.. Appl Environ Microbiol.

[51] Delmotte N, Knief C, Chaffron S, Innerebner G, Roschitzki B (2009). Community proteogenomics reveals insights into the physiology of phyllosphere bacteria.. Proc Natl Acad Sci U S A.

[52] Munkacsi AB, Stoxen S, May G (2008). *Ustilago maydis* populations tracked maize through domestication and cultivation in the Americas.. Proc Biol Sci.

[53] Holst I, Moreno JE, Piperno DR (2007). Identification of teosinte, maize, and tripsacum in Mesoamerica by using pollen, starch grains, and phytoliths.. Proc Natl Acad Sci U S A.

[54] Nabhan GP, Madison D, Nabhan GP (2008). Renewing America's food traditions: saving and savoring the continent's most endangered foods;.

[55] Mauricio RAS, Figueroa JD, Taba S, Reyes ML, Rincón F (2004). Caracterización de accesiones de maíz por calidad de grano y tortilla.. Rev Fitotec Mex.

[56] Gonzalez E, Cardenas JdD, Taba S (2007). Aspectos microestructurales y posibles usos del maiz de acuerdo con su origen geographico.. Rev Fitotech Mex.

[57] Bellon MR, Brush SB (1994). Keepers of maize in Chiapas, Mexico.. Econ Bot.

[58] Gibson RW, Lyimo NG, Temu AEM, Stathers TE, Page WW (2005). Maize seed selection by East African smallholder farmers and resistance to maize streak virus.. Ann Appl Biol.

[59] Saunders M, Kohn LM (2009). Evidence for alteration of fungal endophyte community assembly by host defense compounds.. New Phytol.

[60] Levings CS, Siedow JN (1992). Molecular basis of disease susceptibility in the Texas cytoplasm of maize.. Plant Mol Biol.

[61] Dubos RJ, Schaedler RW (1960). The effect of the intestinal flora on the growth rate of mice, and on their susceptibility to experimental infections.. J Exp Med.

[62] Dominguez-Bello MG, Costello EK, Contreras M, Magris M, Hidalgo G (2010). Delivery mode shapes the acquisition and structure of the initial microbiota across multiple body habitats in newborns.. Proc Natl Acad Sci U S A.

[63] Block CC, Hill JH, McGee DC (1998). Seed transmission of *Pantoea stewartii* in field and sweet corn.. Plant Dis.

[64] Christensen MJ, Voisey CR (2007). The biology of the endophyte/grass partnership..

[65] Pirttilä AM, Laukkanen H, Pospiech H, Myllylä R, Hohtola A (2000). Detection of intracellular bacteria in the buds of Scotch pine (*Pinus sylvestris* L.) by *in situ* hybridization.. Appl Environ Microbiol.

[66] Madmony A, Chernin L, Pleban S, Peleg E, Riov J (2005). *Enterobacter cloacae*, an obligatory endophyte of pollen grains of Mediterranean pines.. Folia Microbiol.

[67] Ela SW, Anderson MA, Brill WJ (1982). Screening and selection of maize to enhance associative bacterial nitrogen fixation.. Plant Physiol.

[68] Ryu C-M, Farag MA, Hu C-H, Reddy MS, Wei H-X (2003). Bacterial volatiles promote growth in *Arabidopsis*.. Proc Natl Acad Sci U S A.

[69] Quadt-Hallmann A, Kloepper JW, Benhamou N (1997). Bacterial endophytes in cotton: mechanisms of entering the plant.. Can J Microbiol.

[70] Nelson EB (2004). Microbial dynamics and interactions in the spermosphere.. Phytopathology.

[71] Chee-Sanford JC, Williams MM, Davis AS, Sims GK (2006). Do microorganisms influence seed-bank dynamics?. Weed Sci.

[72] Puente ME, Li CY, Bashan Y (2009). Endophytic bacteria in cacti seeds can improve the development of cactus seedlings.. Environ Exp Bot.

[73] Torres A, Araújo W, Cursino L, Hungria M, Plotegher F (2008). Diversity of endophytic enterobacteria associated with different host plants.. J Microbiol.

[74] Coutinho TA, Venter SN (2009). *Pantoea ananatis*: an unconventional plant pathogen.. Mol Plant Pathol.

[75] Toth IK, Bell KS, Holeva MC, Birch PRJ (2003). Soft rot erwiniae: from genes to genomes.. Mol Plant Pathol.

[76] Cruz AT, Cazacu AC, Allen CH (2007). *Pantoea agglomerans*-a plant pathogen causing human disease.. J Clin Microbiol.

[77] Feng Y, Shen D, Song W (2006). Rice endophyte *Pantoea agglomerans* YS19 promotes host plant growth and affects allocations of host photosynthates.. Appl Environ Microbiol.

[78] Kloepper J, Leong J, Teintze M, Schroth M (1980). *Pseudomonas* siderophores: A mechanism explaining disease-suppressive soils.. Curr Microbiol.

[79] Glick BR (2005). Modulation of plant ethylene levels by the bacterial enzyme ACC deaminase.. FEMS Microbiol Lett.

[80] Preston GM (2004). Plant perceptions of plant growth-promoting *Pseudomonas*.. Philos Trans R Soc Lond B Biol Sci.

[81] Andreote FD, de Araujo WL, de Azevedo JL, van Elsas JD, da Rocha UN (2009). Endophytic colonization of potato (*Solanum tuberosum* L.) by a novel competent bacterial endophyte, *Pseudomonas putida* strain P9, and its effect on associated bacterial communities.. Appl Environ Microbiol.

[82] Romanovskaia VA, Stoliar SM, Malashenko IR, Dodatko TN (2001). Processes of plant colonization by *Methylobacterium* strains and some bacterial properties.. Mikrobiologiia.

[83] Abanda-Nkpwatt D, Müsch M, Tschiersch J, Boettner M, Schwab W (2006). Molecular interaction between *Methylobacterium extorquens* and seedlings: growth promotion, methanol consumption, and localization of the methanol emission site.. J Exp Bot.

[84] Gai CS, Lacava PT, Quecine MC, Auriac MC, Lopes JRS (2009). Transmission of *Methylobacterium mesophilicum* by *Bucephalogonia xanthophis* for paratransgenic control strategy of citrus variegated chlorosis.. J Microbiol.

[85] Ryu J, Madhaiyan M, Poonguzhali S, Yim W, Indiragandhi P (2006). Plant growth substances produced by *Methylobacterium* spp. and their effect on tomato (*Lycopersicon esculentum* L.) and red pepper (*Capsicum annuum* L.) growth.. J Microbiol Biotechnol.

[86] Lidstrom ME, Chistoserdova L (2002). Plants in the pink: Cytokinin production by *Methylobacterium*.. J Bacteriol.

[87] Mano Y, Omori F, Takamizo T, Kindiger B, Bird R (2006). Variation for root aerenchyma formation in flooded and non-flooded maize and teosinte seedlings.. Plant Soil.

[88] Mano Y, Omori F, Loaisiga CH, Bird RMK (2009). QTL mapping of above-ground adventitious roots during flooding in maize×teosinte (*Zea nicaraguensis*) backcross population.. Plant Root.

[89] de Klerk G-J, van der Krieken W, de Jong J (1999). Review the formation of adventitious roots: New concepts, new possibilities.. In Vitro Cell Dev Biol Plant.

[90] Iltis HH, Doebley JF, M RGN, Pazy B (1979). *Zea diploperennis* (Gramineae): A new teosinte from Mexico.. Science.

[91] Sessitsch A, Coenye T, Sturz AV, Vandamme P, Barka EA (2005). *Burkholderia phytofirmans* sp. nov., a novel plant-associated bacterium with plant-beneficial properties.. Int J Syst Evol Microbiol.

[92] Compant S, Nowak J, Coenye T, Clément C, Ait Barka E (2008). Diversity and occurrence of *Burkholderia* spp. in the natural environment.. FEMS Microbiol Rev.

[93] Long HH, Schmidt DD, Baldwin IT (2008). Native bacterial endophytes promote host growth in a species-specific manner; Phytohormone manipulations do not result in common growth responses.. PLoS ONE.

[94] Raven PH, Franklin ER, Eichhorn SE (1999). Biology of Plants.

[95] Riggs PJ, Chelius MK, Kaeppler SM, Iniguez AL, Triplett EW (2001). Enhanced maize productivity by inoculation with diazotrophic bacteria.. Funct Plant Biol.

[96] Quadt-Hallmann A, Kloepper JW (1996). Immunological detection and localization of the cotton endophyte *Enterobacter asburiae* JM22 in different plant species.. Can J Microbiol.

[97] Asis CA, Adachi K (2004). Isolation of endophytic diazotroph *Pantoea agglomerans* and nondiazotroph *Enterobacter asburiae* from sweetpotato stem in Japan.. Lett Appl Microbiol.

[98] Deepa CK, Dastager SG, Pandey A (2010). Isolation and characterization of plant growth promoting bacteria from non-rhizospheric soil and their effect on cowpea (*Vigna unguiculata* (L.) Walp.) seedling growth.. World J Microbiol Biotechnol.

[99] Gyaneshwar P, Parekh LJ, Archana G, Poole PS, Collins MD (1999). Involvement of a phosphate starvation inducible glucose dehydrogenase in soil phosphate solubilization by *Enterobacter asburiae*.. FEMS Microbiol Lett.

[100] Frank JA, Reich CI, Sharma S, Weisbaum JS, Wilson BA (2008). Critical evaluation of two primers commonly used for amplification of bacterial 16S rRNA genes.. Appl Environ Microbiol.

[101] Gich FB, Amer E, Figueras JB, Charles AA, Balaguer MD (2000). Assessment of microbial community structure changes by amplified ribosomal DNA restriction analysis (ARDRA).. Int Microbiol.

[102] Altschul SF, Gish W, Miller W, Myers EW, Lipman DJ (1990). Basic local alignment search tool.. J Mol Biol.

[103] Junier P, Junier T, Witzel K-P (2008). TRiFLe: a program for in silico T-RFLP analysis with user-defined sequences sets.. Appl Environ Microbiol.

[104] Hall TA (1999). BioEdit: a user-friendly biological sequence alignment editor and analysis program for Windows 95/98/NT.. Nucleic Acids Symposium Series.

[105] Drummond A, Ashton B, Buxton S, Cheung M, Cooper A (2011). Geneious v5.4. 5.4 ed..

[106] Rodriguez H, Gonzalez T, Selman G (2001). Expression of a mineral phosphate solubilizing gene from *Erwinia herbicola* in two rhizobacterial strains.. J Biotechnol.

[107] Hole RC, Singhal RS, Melo JS, D'Souza SF (2004). A rapid plate screening technique for extracellular ribonuclease producing strains.. BARC Newsletter.

[108] Phalip V, Schmitt P, Diviès C (1994). A method for screening diacetyl and acetoin-producing bacteria on agar plates.. J Basic Microbiol.

[109] Bric JM, Bostock RM, Silverstone SE (1991). Rapid in situ assay for indoleacetic acid production by bacteria immobilized on a nitrocellulose membrane.. Appl Environ Microbiol.

[110] Cox CD (1994). Deferration of laboratory media and assays for ferric and ferrous ions.. Methods Enzymol.

[111] Perez-Miranda S, Cabirol N, George-Tellez R, Zamudio-Rivera LS, Fernandez FJ (2007). O-CAS, a fast and universal method for siderophore detection.. J Microbiol Methods.

[112] Soares M, Silva R, Gomes E (1999). Screening of bacterial strains for pectinolytic activity: characterization of the polygalacturonase produced by *Bacillus* sp.. Rev de Microbiol.

[113] Kasana RC, Salwan R, Dhar H, Dutt S, Gulati A (2008). A rapid and easy method for the detection of microbial cellulases on agar plates using gram's iodine.. Curr Microbiol.

